# Fear-of-intimacy-mediated zinc transport is required for *Drosophila* fat body endoreplication

**DOI:** 10.1186/s12915-023-01588-0

**Published:** 2023-04-17

**Authors:** Xiaowen Ji, Jiajia Gao, Tian Wei, Li Jin, Guiran Xiao

**Affiliations:** 1 China Light Industry Key Laboratory of Meat Microbial Control and Utilization, Hefei University of Technology, Hefei, 230009 China; 2 School of Food and Biological Engineering, Hefei University of Technology, Hefei, 230009 China; 3 Department of Toxicology, School of Public Health, Anhui Medical University, Hefei, 230032 China

**Keywords:** FOI, Zinc, Fat body, Endoreplication, JNK, Myc

## Abstract

**Background:**

Endoreplication is involved in the development and function of many organs, the pathologic process of several diseases. However, the metabolic underpinnings and regulation of endoreplication have yet to be well clarified.

**Results:**

Here, we showed that a zinc transporter fear-of-intimacy (*foi*) is necessary for *Drosophila* fat body endoreplication. *foi* knockdown in the fat body led to fat body cell nuclei failure to attain standard size, decreased fat body size and pupal lethality. These phenotypes could be modulated by either altered expression of genes involved in zinc metabolism or intervention of dietary zinc levels. Further studies indicated that the intracellular depletion of zinc caused by *foi* knockdown results in oxidative stress, which activates the ROS-JNK signaling pathway, and then inhibits the expression of Myc, which is required for tissue endoreplication and larval growth in *Drosophila*.

**Conclusions:**

Our results indicated that FOI is critical in coordinating fat body endoreplication and larval growth in *Drosophila*. Our study provides a novel insight into the relationship between zinc and endoreplication in insects and may provide a reference for relevant mammalian studies.

**Supplementary Information:**

The online version contains supplementary material available at 10.1186/s12915-023-01588-0.

## Background

Endoreplication, known as endocycles, endoreduplication, or endopolyploidization, is a cell cycle variant in which cellular growth and DNA replication occur without mitosis [1]. Endoreplication is essential for the development and function of many organs in animals and plants. Besides, endoreplication occurs in many diseases to drive morphologic growth, cell fate, and physiological function [2, 3]. The metabolic underpinnings and regulation of endoreplication need to be better clarified.

Insect fat body (analogous to vertebrate adipose tissues and liver) is an interchanging center in regulating development and behavior, such as larval growth, circadian clock, and longevity [4–7]. Besides, the fat body has emerged as a fascinating model for studying metabolic disorders and immune diseases, including obesity, diabetes, inflammation, and so on [8–11]. It has been reported that insect fat body cells undergo mitosis during embryogenesis, endoreplication during the larval stages, and remodeling during metamorphosis [5]. Therefore, understanding insect fat body biology is essential for human developmental disease studies [5]. Many genes and signaling pathways have been reported to be involved in the development of *Drosophila* *melanogaster* fat body, and *Drosophila* has gained appreciation as a valuable model for studying endoreplication [12–15].

Zinc is one micronutrient that contributes to various physiological processes in most organisms [16, 17]. Two large families of zinc transporters mediate zinc homeostasis, the SLC39A or Zip (which generally function in zinc influx from the extracellular medium or vesicular organelles into the cytoplasm) and the SLC30A or ZnT (which mediate zinc efflux or sequestration into organelles/vesicles from of the cytoplasm) [18, 19]. *Drosophila* fear-of-intimacy (FOI) is a zinc transporter localized to the cell plasma membrane [20], which shares the highest overall homology with mammalian Zip6 and Zip10 [18]. Mammalian Zip6 and Zip10 are involved in several important physiological processes, such as macrophage survival, epidermal development, and oocyte development [17, 21–24]. *Drosophila* FOI has been reported to be involved in gonad and trachea morphogenesis, glial cell migration, myogenesis, cell dissociation, and so on [25–29]. We have long been interested in understanding the physiological function of zinc in vivo. According to the FlyAtlas database [30], *foi* is enrichment in the fat body. Our previous work showed that when *foi* was knocked down with *Cg*-Gal4, a driver specifically expressing the activator Gal4 in the fat body and hemocytes at all stages of development [13, 31], *Drosophila* displayed developmental arrest at the pupal stage (died before eclosion), this suggests that FOI involves the early development of *Drosophila* [29]. However, the role of FOI in the early development of the *Drosophila* fat body has yet to be investigated in depth.

In this study, we showed that *Drosophila* *foi* RNAi in the fat body led to developmental defects and growth-retarded phenotypes, which can be ameliorated by dietary zinc supplementation or the genetic modulation of other zinc transporters. These phenotypes resulted from defects in fat body cell endoreplication. Further studies showed that the zinc deficiency in *foi* RNAi results in the accumulation of ROS and activation of the JNK signaling pathway, which could inhibit the expression of Myc in the fat body and then lead to defects in endoreplication and larval growth. In summary, our findings reveal a novel role of FOI in endoreplication-related growth during larval stages in *Drosophila* and provide new insights into understanding insect fat body development.

## Results

### *Drosophila* FOI is required for the proper formation of the fat body

**Fig. 1:**
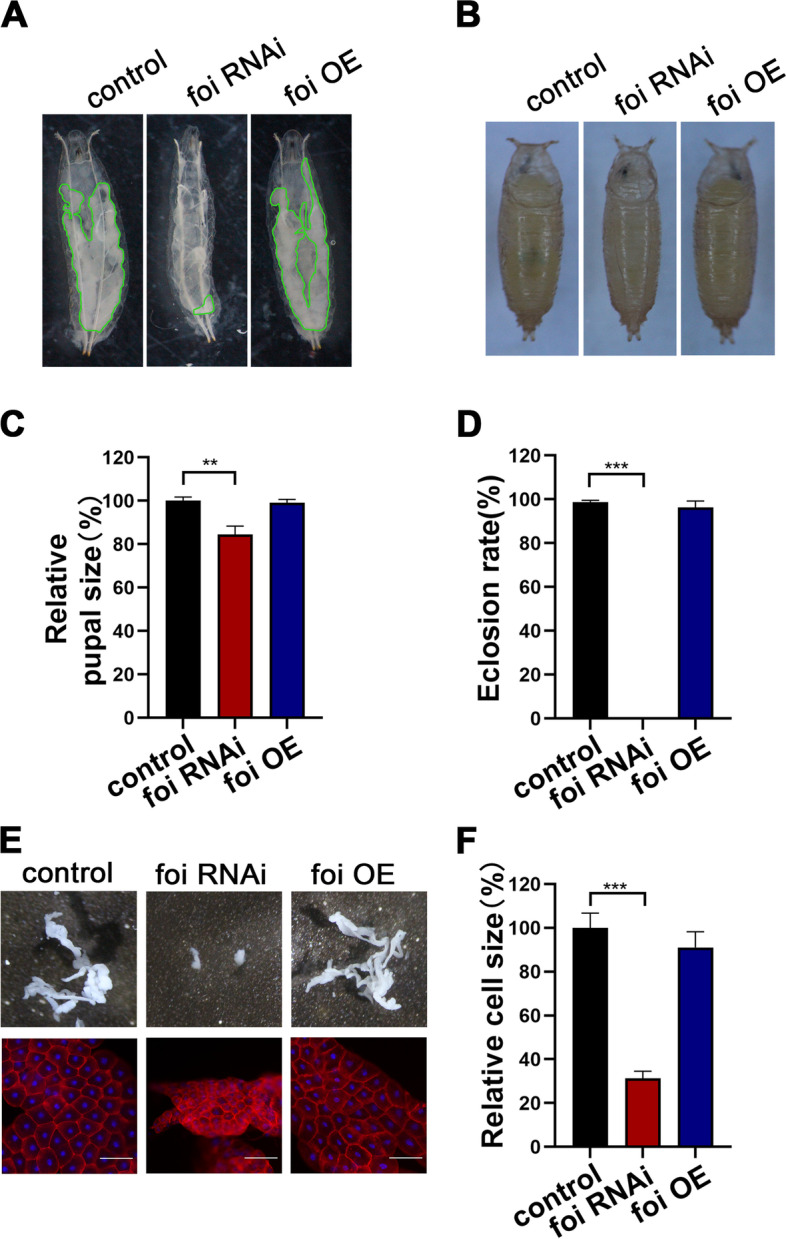
*Drosophila * FOI is required for larval fat body development. **A** Morphological analysis indicated a decrease in larvae size and a significant fat body developmental defect of *foi* RNAi third instar larvae, whereas no significant difference was observed for *foi* OE. The green line marks present the fat body tissues. *n* = 6 replicates per group. **B** Morphological analysis indicated a decrease in pupal size of *foi* RNAi compared with control, whereas no significant difference was observed for *foi* OE. *n* = 10 replicates per group. **C** Quantitative measurement of (**B**) *n* = 10 replicates per group. **D** Eclosion rate of *Cg*-Gal4 > *foi* RNAi was reduced, whereas no significant difference was observed for *Cg*-Gal4 > *foi* OE. *n* = 50–70 larvae per vial, *n* = 6 vials per experimental group. **E** Knockdown of *foi* led to reduced fat body size and cell size in the fat body, whereas no significant difference was observed for *Cg*-Gal4 > *foi* OE. *n* = 6 replicates per group. Scale bar, 100 μm. **F** Quantitative measurement of the fat body cell sizes in (**E**). *n* = 6 replicates per group. Genotypes used in (**A**-**F**) were *Cg*-Gal4 > *w*^*1118*^ (control), *Cg*-Gal4 > *foi* RNAi or *Cg*-Gal4 > *foi* OE. All values are presented as mean ± SEM of the biological replicates. **p < 0.01, ***p < 0.001; two-tailed Student’s t-test. OE, overexpression

We modulated *foi* expression in the larval fat body using the UAS-Gal4 binary system to explore the function of FOI in the fat body. The *Cg* (collagen type IV) promoter fused with Gal4 (*Cg*-Gal4) was used to modulate *foi* in the fat body specifically [32]. *Cg*-Gal4 is strongly expressed at all stages of development [32, 33]. Fat body tissues occupied the entire abdominal cavity of third instar larvae in control and *foi* overexpression (OE) (Fig. 1A). Still, most fat body tissues disappeared in *foi* RNAi larvae. Even the midgut was visible directly underneath the epidermis due to a lack of fat body surrounding (Fig. 1A). Analysis of organismal size revealed that *foi* RNAi animals develop with a significantly smaller size throughout development than control animals (Fig. 1A-C). The pupal size of *foi* RNAi dropped to about 84% of the control (Fig. 1B-C). Besides, *foi* knockdown in the fat body resulted in a developmental arrest at the pupal stage (Fig. 1D and Additional file 1: Fig. S1A).

To assess whether the differences in body size measured in *foi* RNAi animals reflected changes in the size of organs, we measured the organ size and cell size of the fat body in third-instar larvae. The results showed that the fat body size was significantly smaller in *foi* RNAi larvae (Fig. 1E and Additional file 1: Fig. S1B). The cell size of *foi* RNAi larvae reduced to about 31% compared to the control (Fig. 1E-F and Additional file 1: Fig. S1B). No obvious phenotype was observed in *foi* OE larvae (Fig. 1A-F). Two *foi* RNAi flies, V10102# (used in the main text and referred to as *foi* RNAi) and V330251# (referred to as *foi* RNAi 2# in the Supplement Material), exhibited largely similar phenotypes, except that the phenotypes of the *foi* RNAi line were more severe than those of the *foi* RNAi 2# line. Together, these results revealed that the expression of FOI is essential for the early development of the *Drosophila* fat body.

### Diet zinc intervention could modulate the developmental defects caused by *foi* knockdown

**Fig. 2:**
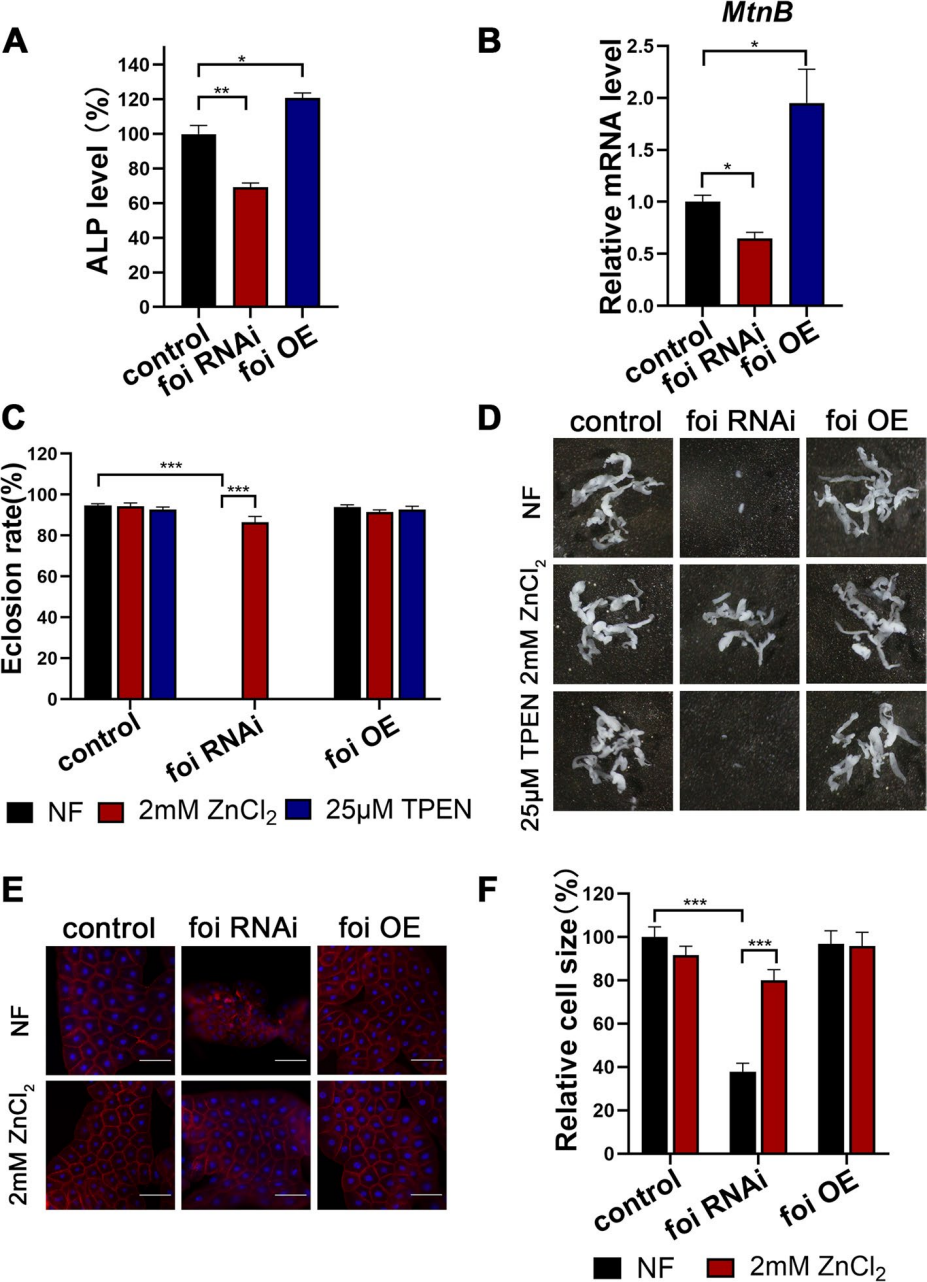
Knockdown of *foi* leads to zinc deficiency in the fat body, and dietary zinc intervention modified a spectrum of phenotypes associated with *foi* knockdown. **A** Alkaline phosphatase (ALP) activity was significantly decreased in the fat body of *Cg*-Gal4 > *foi* RNAi third instar larvae and slightly increased in *Cg*-Gal4 > *foi* OE larvae. *n* = 40 fat bodies per group. Quantification from three independent experiments. All values are presented as mean ± SEM and included in Additional file 2. **B** Q-RT-PCR analysis of *MtnB* transcript levels showed that the expression of *MtnB* in the fat body was down-regulated in *Cg*-Gal4 > *foi* RNAi and upregulated in *Cg*-Gal4 > *foi* OE third instar larvae. RNA was made from the third instar larvae’s fat body. *rp49* was used as control. *n* = 40 fat bodies per group. Quantification from three independent experiments. All values are presented as mean ± SEM and included in Additional file 2. **C** The eclosion rate of *Cg*-Gal4 > *foi* RNAi larvae could be rescued by dietary zinc supplementation. *Cg*-Gal4 was crossed to wild-type, *foi* RNAi or *foi* OE flies on juice-agar plates. Newly hatched progeny were transferred to normal food (NF) or food supplemented with zinc (2 mM ZnCl_2_) or zinc chelator (25 μM N, N, N’, N’-tetrakis (2-pyridylmethyl) ethylenediamine [TPEN]). Percentages of flies that eclosed to adults were counted. *n* = 50–70 larvae per vial, *n* = 6 vials per experimental group. **D** The reduced fat body size of *Cg*-Gal4 > *foi* RNAi larvae could be rescued by dietary zinc supplementation (2 mM ZnCl_2_) but exacerbated by dietary zinc depletion (25 μM TPEN) in food. *n* = 6 replicates per group. **E** The reduced fat body cell size of *Cg*-Gal4 > *foi* RNAi larvae could be rescued by dietary zinc supplementation (2 mM ZnCl_2_) and exacerbated by dietary zinc depletion (25 μM TPEN) in food. *n* = 6 replicates per group. Scale bar, 100 μm. **F** Quantitative measurement of the fat body cell sizes in (**E**). Genotypes used in (**A**-**F**) were *Cg*-Gal4 > *w*^*1118*^ (control), *Cg*-Gal4 > *foi* RNAi or *Cg*-Gal4 > *foi* OE. *n* = 6 replicates per group in (**C**-**F**). Results are presented as mean ± SEM of the biological replicates. *p < 0.05, **p < 0.01, ***p < 0.001; two-tailed Student’s t-test. OE, overexpression

Previously, we reported that *foi* modulates zinc homeostasis in the fat body cells at the mid-instar transition midway through the third larval instar [29]. It remains unknown whether it is involved in zinc homeostasis at an earlier development stage. Alkaline phosphatase (ALP) activity has been used as a sensitive indicator of intracellular zinc levels [34, 35]. As shown in Fig. 2A, ALP activity was significantly reduced (~ 31%) in the fat body of *foi* RNAi larvae, compared to control larvae, indicating that zinc levels were reduced in the fat body of *foi* RNAi. In comparison, *foi* OE exhibited somewhat elevated ALP activity (~ 21%). *Metallothionein B* (*MtnB*) expression has been previously reported as sensitive to zinc increase in cells and is considered a sensitive indicator of intracellular zinc levels [16]. Knockdown of *foi* in fat body showed a significant reduction of *MtnB* mRNA levels (~ 35%). In contrast, *foi* OE showed elevated *MtnB* mRNA levels (~ 95%) (Fig. 2B). These results indicated that FOI modulates zinc homeostasis at an earlier development stage in the fat body of *Drosophila*.

We subsequently tested whether dietary zinc intervention with zinc supplementation or depletion could affect the developmental defects caused by *foi* knockdown. Dietary zinc supplementation (2 mM ZnCl_2_) could rescue the survival rate of *Cg-*Gal4 > *foi RNAi* from 0 to 87% (Fig. 2C) and *Cg-*Gal4 > *foi RNAi 2#* from 16 to 93% (Additional file 1: Fig. S1A). Consistently, the fat body developmental defects caused by *foi* RNAi could be significantly rescued or exacerbated by dietary zinc supplementation (addition of 2 mM ZnCl_2_) or zinc depletion (addition of 25 μM zinc chelator N, N, N′, N′-tetrakis (2-pyridylmethyl) ethylenediamine [TPEN]), respectively (Fig. 2D, Additional file 1: Figs. S1B and S2A-B). As mentioned, *foi* RNAi was lethal at the pupal stage, but *foi* RNAi cultured on TPEN were lethal at the larval stage (Additional file 1: Fig. S2A). Besides, the fat body size of *foi* RNAi was also aggravated by TPEN (Additional file 1: Fig. S2B). As a control, 25 μM TPEN did not affect the fat body development of the wild type (Additional file 1: Fig. S3). We then analyzed the effects of zinc on cell size in the fat body of third instar larvae. Consistent with the results described above, zinc supplementation significantly rescued the cell size of the fat body in *foi* RNAi from 38 to 80% (Fig. 2E-F and Additional file 1: Fig. S1B). Notably, TPEN so much aggravated the fat body developmental defects caused by *foi* RNAi that it was difficult to get a piece of complete fat body (Fig. 2D). These results indicate that the fat body developmental defects caused by *foi* RNAi could be restored by dietary zinc supplementation.

### The developmental defects caused by *foi* knockdown arise from cytosolic zinc reduction

**Fig. 3:**
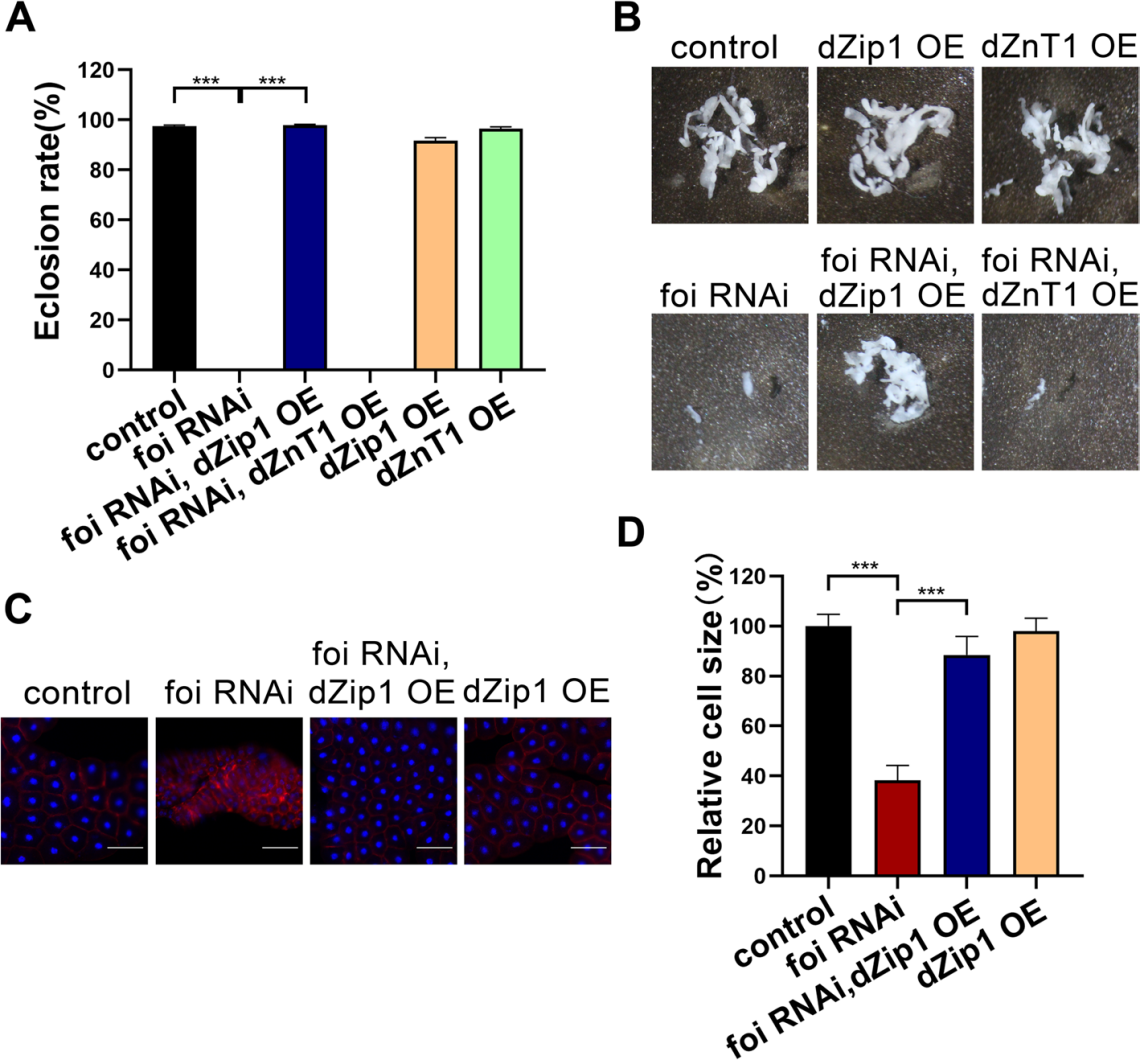
Modulation of zinc transporters altered the developmental defects of *foi* RNAi. **A** The eclosion defect of *Cg*-Gal4 > *foi* RNAi larvae was partially rescued by *dZip1* OE*. n* = 50–70 larvae per vial, *n* = 6 vials per experimental group. **B** The reduced fat body size of *Cg*-Gal4 > *foi* RNAi larvae was partially rescued by *dZip1* OE and exacerbated by *dZnT1* OE. *n* = 6 replicates per group. Genotypes used in (**A**-**B**) were *Cg*-Gal4 > *w*^*1118*^ (control), *Cg*-Gal4 > *foi* RNAi, *Cg*-Gal4 > *foi* RNAi; *dZip1* OE, *Cg*-Gal4 > *dZnT1* OE; *foi* RNAi, *Cg*-Gal4 > *dZip1* OE, *Cg*-Gal4 > *dZnT1* OE. **C** The reduced fat body cell size of *Cg*-Gal4 > *foi* RNAi larvae was significantly rescued by *dZip1* OE. *n* = 6 replicates per group. Scale bar, 100 μm. **D** Quantitative measurement of the fat body cell sizes in (**C**). *n* = 6 replicates per group. Genotypes used in (**C**-**D**) were *Cg*-Gal4 > *w*^*1118*^ (control), *Cg*-Gal4 > *foi* RNAi, *Cg*-Gal4 > *foi* RNAi; *dZip1* OE, *Cg*-Gal4 > *dZip1* OE. *n* = 6 replicates per group. Data are presented as mean ± SEM of the biological replicates. ***p < 0.001; two-tailed Student’s t-test. OE, overexpression

The above data suggest that the effect of FOI on fat body development is related to zinc. If the function of FOI in the fat body is mediated by zinc influx into the cytosol, the defects caused by *foi* RNAi could be modulated by other zinc transporters. Two zinc transporters with similar subcellular distributions to FOI were chosen in the rescue experiments. dZnT1 (ZnT63C, encoded by CG17723) is a ZnT transporter functioning in zinc efflux [71], and dZip1 (Zip42C.1, encoded by CG9428) is a Zip transporter mediating zinc influx [73]. The retarded growth and lethal at pupal stage phenotypes of *foi* RNAi were almost fully rescued by *dZip1* OE while exacerbated by *dZnT1* OE (Fig. 3A, Additional file 1: Figs. S1C and S2C-D). The fat body developmental defects caused by *foi* RNAi were also rescued by *dZip1* OE while exacerbated by *dZnT1* OE (Fig. 3B and Additional file 1: Figs. S1D and S2D). As mentioned above, *foi* RNAi was lethal at the pupal stage, but *dZnT1* OE; *foi* RNAi was lethal at the larval stage (Additional file 1: Fig. S2C). Besides, the quantitative measurement of the fat body size confirmed that fat body developmental defects of *foi* RNAi were aggravated by *dZnT1* OE (Additional file 1: Fig. S2D). Consistently, *dZip1* OE significantly rescued the fat body cell size of *foi* RNAi, with a 50% increase (Fig. 3C-D and Additional file 1: Fig. S1D). Notably, *dZnT1* OE so much aggravated the fat body developmental defects caused by *foi* RNAi that it was difficult to get a piece of the complete fat body (Fig. 3B), so these data could not be shown here (Fig. 3C-D). These results indicate that cytosolic zinc accumulation significantly rescued, whereas cytosolic zinc reduction exacerbated the fat body developmental defects caused by *foi* knockdown in this tissue.

To investigate whether the defects caused by *foi* knockdown in the fat body are related to other trace metal elements, we tested the effect of iron supplementation or depletion on the developmental defects caused by *foi* knockdown (Additional file 1: Fig. S1E-F). Neither dietary iron supplementation by ferric ammonium citrate (FAC) nor dietary iron restriction with iron chelator bathophenanthrolinedisulfonic acid disodium (BPS) showed any effect on the growth retardation or fat body developmental defects of *foi* RNAi larvae (Additional file 1: Fig. S1E-F), indicating that the effect of *foi* knockdown on fat body development is specifically related to zinc. All these results suggested that the zinc dyshomeostasis in the cytoplasm is responsible for the developmental and growth defects in *foi* RNAi larvae.

### *Drosophila* FOI is required for DNA endoreplication in fat body cells

**Fig. 4:**
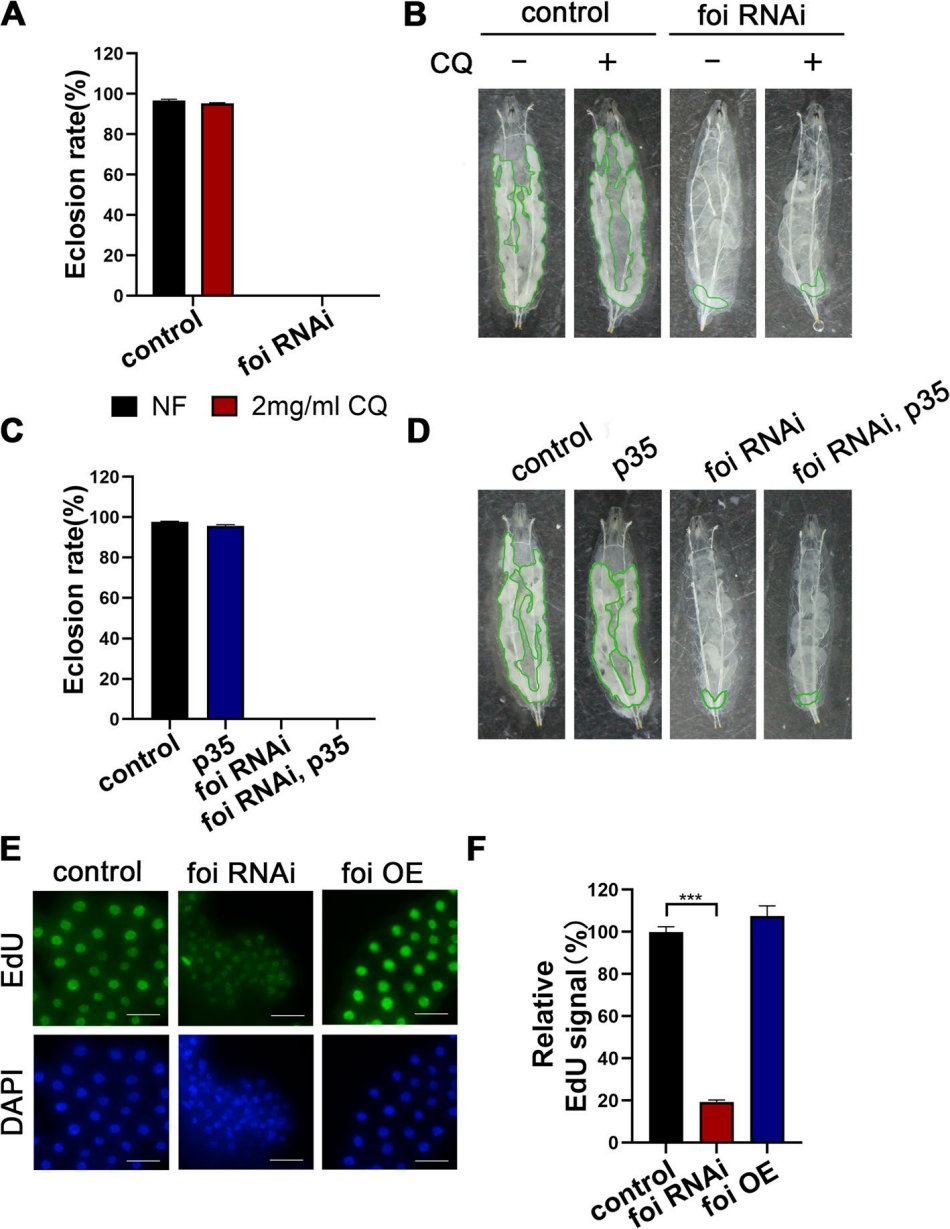
The developmental defect of *foi* RNAi is related to DNA endoreplication. **A** Autophagy inhibitor chloroquine (CQ) did not affect the eclosion defect of *Cg*-Gal4 > *foi* RNAi larvae. *n* = 50–70 larvae per vial, *n* = 6 vials per experimental group. **B** The fat body developmental defect of *Cg*-Gal4 > *foi* RNAi larvae was not rescued by CQ. Green line marks present the fat body tissues. *n* = 6 replicates per group. Genotypes used in (**A**-**B**) were *Cg*-Gal4 > *w*^*1118*^ (control), *Cg*-Gal4 > *foi* RNAi. **C** The eclosion defect of *Cg*-Gal4 > *foi* RNAi larvae was not affected by the apoptosis inhibitor *p35. n* = 50–70 larvae per vial, *n* = 6 vials per experimental group. **D** The fat body developmental defect of *Cg*-Gal4 > *foi* RNAi larvae showed no significant change in the presence of the apoptosis inhibitor *p35*. The green line marks present the fat body tissues. *n* = 6 replicates per group. Genotypes used in (**C**-**D**) were *Cg*-Gal4 > *w*^*1118*^ (control), *Cg*-Gal4 > *p35*, *Cg*-Gal4 > *foi* RNAi, *Cg*-Gal4 > *p35*; *foi* RNAi. **E** Nuclei double stained with 4’,6-diamidino-2-phenylindole (DAPI, blue) and 5-Ethynyl-2'-deoxyuridine (EdU, green) showed that the nuclei of *Cg*-Gal4 > *foi* RNAi display abnormal shape, smaller size and weaker fluorescence signals compared with control nuclei (*Cg*-Gal4 > *w*^*1118*^). *n* = 6 replicates per group. Scale bar, 50 μm. **F** The replication signals of EdU-positive cells in control, *foi* RNAi, or *foi* OE larvae fat body tissues were determined for the experiment in (**E**). *n* = 6 replicates per group. (control, *n* = 88; *Cg*-Gal4 > *foi* RNAi, *n* = 97; *Cg*-Gal4 > *foi* OE, *n* = 128 nuclei). Genotypes used in (**E**–**F**) were *Cg*-Gal4 > *w*^*1118*^ (control), *Cg*-Gal4 > *foi* RNAi, *Cg*-Gal4 > *foi* OE. Data are represented as mean ± SEM of the biological replicates. ***p < 0.001; two-tailed Student’s t-test. OE, overexpression

As mentioned above, fat body tissues become smaller in *foi* RNAi larvae. Cell death, such as autophagy and apoptosis, is reported to inhibit cell growth in *Drosophila* fat body [14, 36], so we wondered whether the fat body developmental defects caused by *foi* RNAi are related to autophagy or apoptosis. Chloroquine (CQ) is an autophagy inhibitor that blocks the fusion of autophagosomes with lysosomes [37]. However, neither the decreased eclosion rate (Fig. 4A) nor fat body developmental defects (Fig. 4B) of *foi* RNAi larvae could be rescued by CQ supplementation in food. This indicates that the fat body developmental defects caused by *foi* RNAi are unrelated to autophagy. *Baculovirus p35* is well known as an inhibitor of apoptosis [38–40]. We subsequently tested the effects of the anti-apoptotic gene on the phenotypes of *foi* RNAi larvae. The results showed that the developmental defects of *foi* RNAi were not affected by *Baculovirus p35* (Fig. 4C-D). This suggests that apoptosis is not involved in the effect of *foi* RNAi on the fat body developmental defects. Larval growth involves both cytoplasmic growth and DNA endoreplication [41]. It is known that the fat body is an endoreplication tissue, where cellular growth and DNA replication occur in the absence of cell division during the larval stage [15, 31]. Endoreplication defects make this tissue fail to attain standard size along with the smaller nuclear size and reduced DNA levels, which results in growth defects [15, 31]. To investigate the nuclear size and the levels of DNA synthesis of fat body cells, we used 5-Ethynyl-2'-deoxyuridine (EdU) to label S-phase nuclei [31, 42]. The nuclei of fat body cells in *foi* RNAi larvae were much smaller than control (Fig. 4E), consistent with the reduction in cell size. Moreover, the EdU signal in nuclei was decreased in *foi* RNAi compared to the control, indicating decreased DNA levels in these cells (Fig. 4E-F). Based on the above results, that is, the pupae growth arrest, the fat body developmental defects, the reduced rate of cellular growth, as well as the decreased nuclei size and DNA replication of fat body cells, it is reasonable to conclude that FOI is essential for DNA endoreplication in fat body cells.

### Myc plays a key role in FOI’s function on DNA endoreplication

**Fig. 5:**
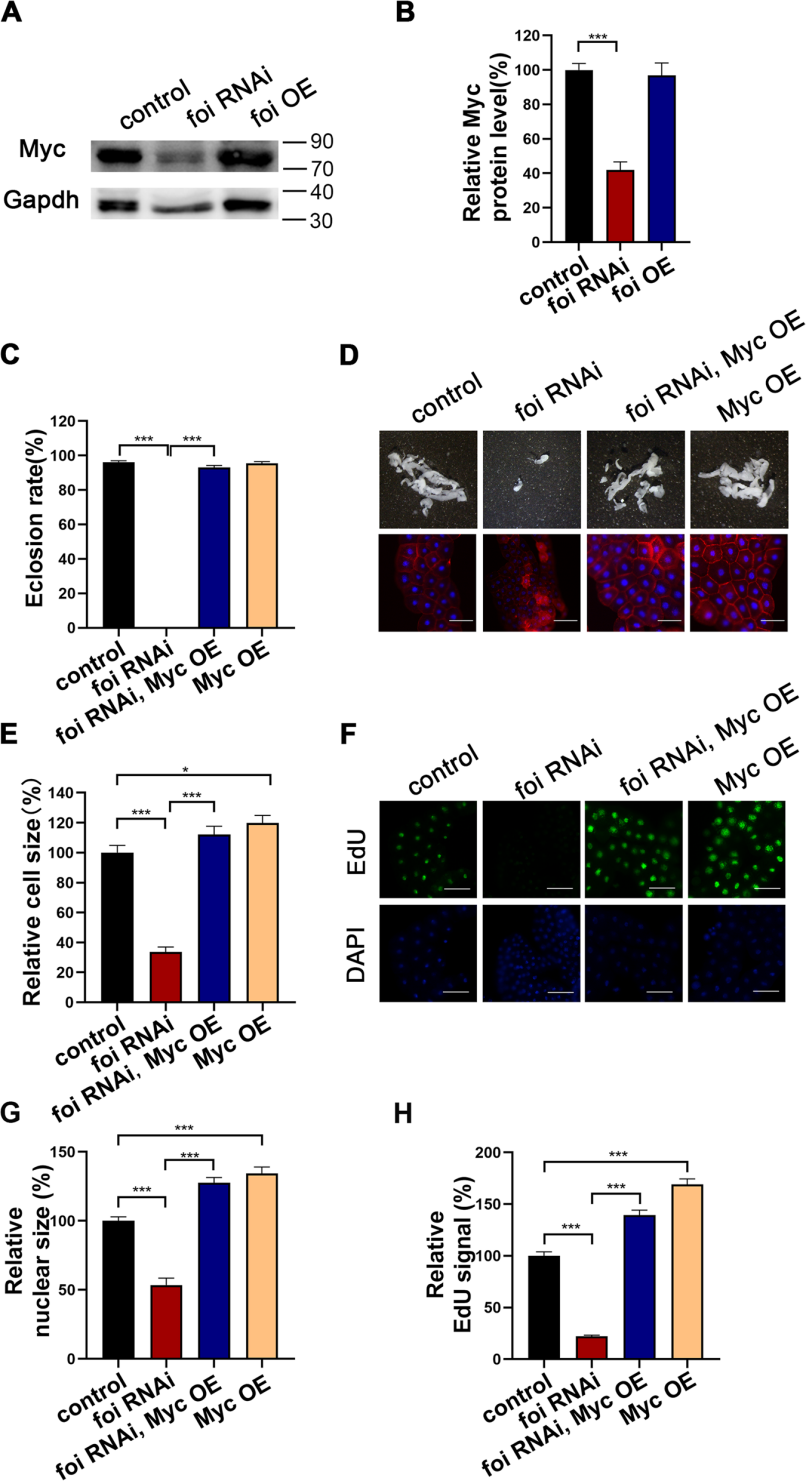
The growth arrest, fat body developmental defects and blocked endoreplication in *foi* RNAi were restored by *Myc* overexpression. **A** Western blot analysis shows that Myc synthesized in the fat body decreased when *foi* was knocked down. Gapdh was used as the loading control. *n* = 40 fat bodies per group. **B** Quantitative measurement of (**A**). Quantification from three independent experiments. Genotypes used in (**A**-**B**) were *Cg*-Gal4 > *w*^*1118*^ (control), *Cg*-Gal4 > *foi* RNAi, *Cg*-Gal4 > *foi* OE. All values are presented as mean ± SEM and included in Additional file 2. **C** The eclosion defect of *Cg*-Gal4 > *foi* RNAi was almost fully rescued by *Myc* OE. *n* = 50–70 larvae per vial, *n* = 6 vials per experimental group. **D** The smaller fat body size and cell size of *Cg*-Gal4 > *foi* RNAi were significantly rescued by *Myc* OE. *n* = 6 replicates per group. Scale bar, 100 μm. **E** Quantitative measurement of the fat body cell sizes in (**D**). *n* = 6 replicates per group. **F** The endoreplication defects observed in *Cg*-Gal4 > *foi* RNAi fat body nuclei were significantly rescued by *Myc* OE. *n* = 6 replicates per group. Scale bar, 100 μm. **G** Quantitative measurement of the nuclei size in (**F**). (control, *n* = 64; *Cg*-Gal4 > *foi* RNAi, *n* = 125; *Cg*-Gal4 > *Myc* OE; *foi* RNAi, *n* = 73; *Cg*-Gal4 > *Myc* OE, *n* = 86 nuclei). **H** Quantitative measurement of the replication signals in (**F**). (control, *n* = 42; *Cg*-Gal4 > *foi* RNAi, *n* = 91; *Cg*-Gal4 > *Myc* OE; *foi* RNAi, *n* = 82; *Cg*-Gal4 > *Myc* OE, *n* = 78 nuclei). Genotypes used in (**C**-**H**) were *Cg*-Gal4 > *w*^*1118*^ (control), *Cg*-Gal4 > *foi* RNAi, *Cg*-Gal4 > *Myc* OE*; foi* RNAi*, Cg*-Gal4 > *Myc* OE. Data are represented as mean ± SEM of the biological replicates. *p < 0.05, ***p < 0.001; two-tailed Student’s t-test. OE, overexpression

*Drosophila diminutive (dm)* gene encodes Myc, a homologous transcription factor to vertebrate Myc proto-oncogene. It contributes to cell growth, competition, and regenerative proliferation [43]. Notably, Myc has been reported to regulate larval development and endoreplication in *Drosophila* endoreplication tissues, including the fat body and salivary gland [15, 42]. Considering the fat body developmental defects of *foi* RNAi by using *Cg*-Gal4 phenocopied *dm* deletion [15], we subsequently examined whether the effect of *foi* on the DNA endoreplication was mediated by Myc. As shown in Fig. 5A-B, Additional file 1: Fig. S4A and Additional file 3, the expression of Myc was significantly downregulated in *foi* RNAi (decreased by 58%) and *foi* RNAi 2# (reduced by 48%) in comparison to the control. These data suggested that *foi* may act as a positive modulator of Myc.

To further investigate whether Myc is involved in the effect of *foi* on DNA endoreplication, *Myc* was overexpressed in *foi* RNAi larvae. Consistent with our hypothesis, the growth arrest and developmental defects of *foi* RNAi larvae could be almost fully restored by *Myc* OE, including the decreased eclosion rate (Fig. 5C and Additional file 1: Fig. S4B), the smaller fat body (Fig. 5D and Additional file 1: Fig. S4C), the reduced cell size (Fig. 5D and E), the reduced nuclei size (Fig. 5F and G) and the decreased DNA replication of fat body cells (Fig. 5F and H). Altogether, these data suggested that Myc depletion was responsible for the DNA endoreplication defects in the fat body cells of *foi* RNAi.

### The regulation of *foi* on Myc expression depends on the c-Jun N-terminal kinase (JNK) signaling pathway

**Fig. 6:**
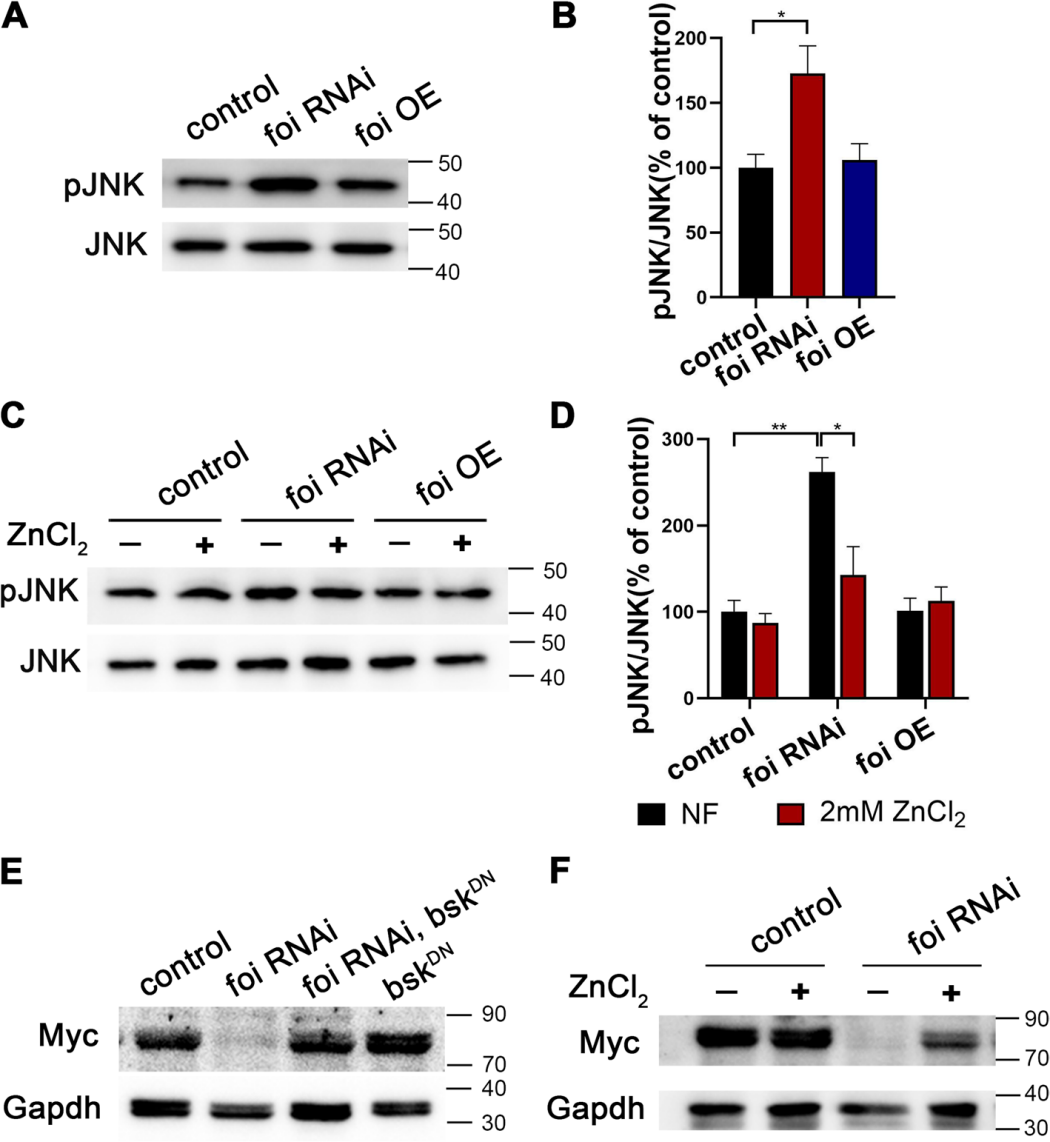
The decreased expression of Myc in *foi* RNAi is mediated by the JNK signaling pathway. **A** Western blot analysis shows that expression of phosphorylated JNK (pJNK) was activated in *foi* RNAi larvae in comparison to that of control. *n* = 40 fat bodies per group. **B** Quantitative measurement of (**A**). Quantification from three independent experiments. All values are presented as mean ± SEM and included in Additional file 2. **C** The increased expression of pJNK in *Cg*-Gal4 > *foi* RNAi larvae fat body was partially suppressed by dietary zinc supplementation. *n* = 40 fat bodies per group. **D** Quantitative measurement of (**C**). Quantification from three independent experiments. Genotypes used in (**A**-**D**) were *Cg*-Gal4 > *w*^*1118*^ (control), *Cg*-Gal4 > *foi* RNAi, *Cg*-Gal4 > *foi* OE. All values are presented as mean ± SEM and included in Additional file 2. **E** The decreased expression of Myc in *foi* RNAi larvae fat body could be significantly restored by JNK signaling inhibition, which is achieved by expression of a dominant negative form of the *basket* (*bsk*^*DN*^). *n* = 40 fat bodies per group. Genotypes were *Cg*-Gal4 > *w*^*1118*^ (control), *Cg*-Gal4 > *foi* RNAi, *Cg*-Gal4 > *bsk*^*DN*^*; foi* RNAi*, Cg*-Gal4 > *bsk*^*DN*^. **F** Dietary zinc supplementation significantly increased Myc expression in the fat body of *Cg*-Gal4 > *foi* RNAi larvae. *n* = 40 fat bodies per group. Genotypes were *Cg*-Gal4 > *w*^*1118*^ (control), *Cg*-Gal4 > *foi* RNAi. Data are represented as mean ± SEM of the biological replicates. *p < 0.05, **p < 0.01; two-tailed Student’s t-test. OE, overexpression

C-Jun N-terminal kinase (JNK) signaling pathway has been reported to regulate endoreplication during *Drosophila* salivary gland development [44], so we wondered whether the effect of *foi* RNAi on endoreplication is mediated by the JNK signaling pathway. Western blot analysis showed that the phosphorylated JNK (pJNK) was significantly upregulated in *foi* RNAi in compared to the control (Fig. 6A-B, Additional file 1: Fig. S4A and Additional file 3). This suggested that *foi* RNAi markedly activated the JNK signaling in *Drosophila* fat body. As we mentioned before, the developmental defects of *foi* RNAi could be rescued well by dietary zinc supplementation. Then we tested whether the activated JNK signaling in *foi* RNAi is related to zinc depletion. Interestingly, the results showed that the JNK activation of *foi* RNAi was suppressed by dietary zinc supplementation (Fig. 6C-D, Additional file 3). No obvious change of pJNK was observed between *foi* OE and control. Furthermore, the decreased level of Myc in *foi* RNAi could be significantly restored by JNK inhibition, which is achieved by ectopic expression of a dominant negative form of the *basket (bsk*^*DN*^) [45] (Fig. 6E, Additional file 3) or dietary zinc supplementation (Fig. 6F, Additional file 3).

**Fig. 7:**
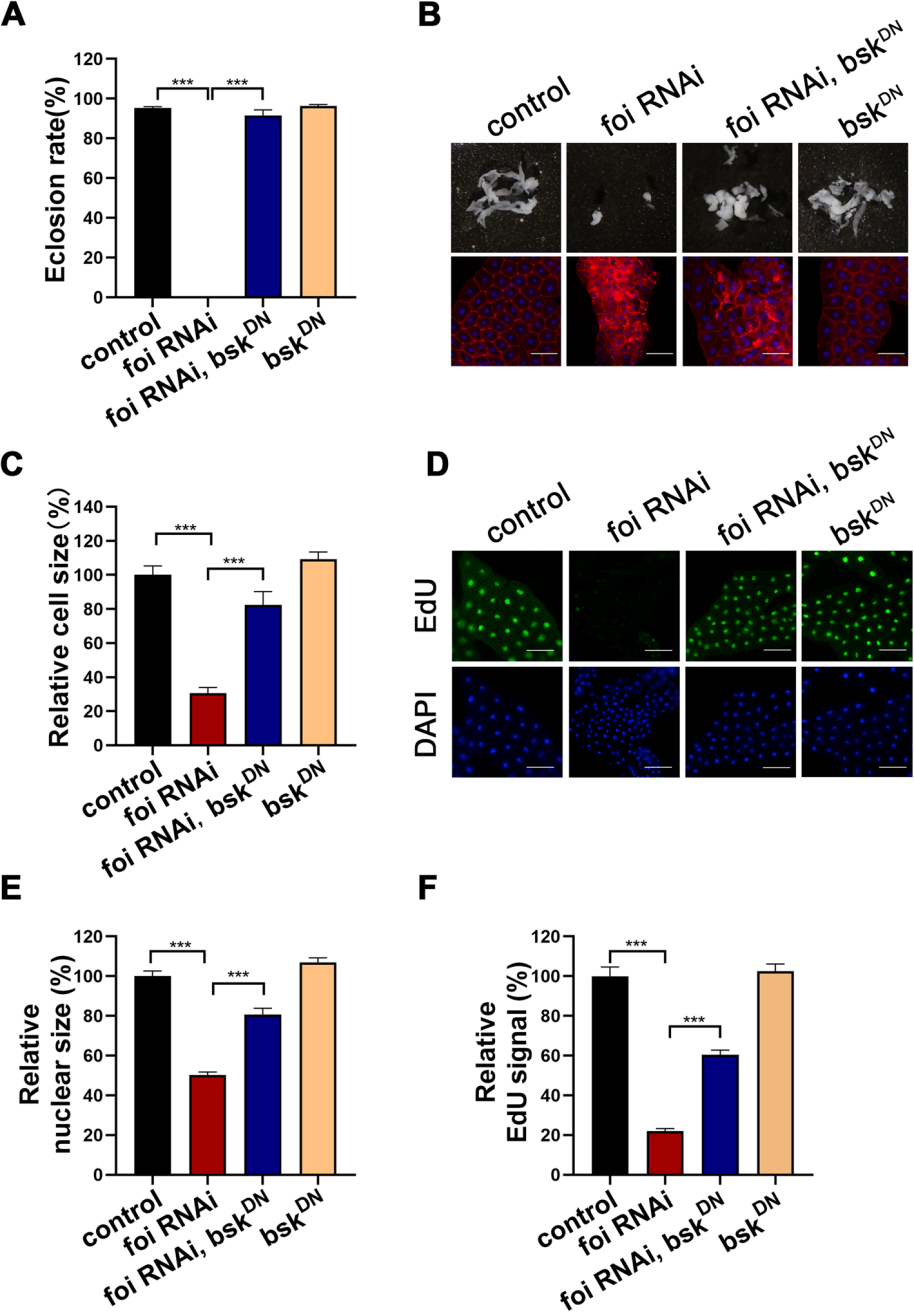
The growth arrest, fat body developmental defects and blocked endoreplication in *foi* RNAi could be rescued by JNK signaling inhibition. **A** The eclosion defect of *Cg*-Gal4 > *foi* RNAi was almost fully rescued by *bsk*^*DN*^. *n* = 50–70 larvae per vial, *n* = 6 vials per experimental group. **B** The smaller fat body size and cell size of *Cg*-Gal4 > *foi* RNAi were rescued very well by *bsk*^*DN*^. *n* = 6 replicates per group. Scale bar, 100 μm. **C** Quantitative measurement of the fat body cell sizes in (**B**). **D** The endoreplication defects observed in *Cg*-Gal4 > *foi* RNAi fat body nuclei were significantly rescued by *bsk*^*DN*^. *n* = 6 replicates per group. Scale bar, 100 μm. **E** Quantitative measurement of the nuclei size in (**D**). (control, *n* = 141; *Cg*-Gal4 > *foi* RNAi, *n* = 108; *Cg*-Gal4 > *bsk*^*DN*^;  *foi* RNAi, *n* = 51; *Cg*-Gal4 > *bsk*^*DN*^, *n* = 100 nuclei). **F** Quantitative measurement of the replication signals in (**D**). (control, *n* = 100; *Cg*-Gal4 > *foi* RNAi, *n* = 106; *Cg*-Gal4 > *bsk*^*DN*^;  *foi* RNAi, *n* = 96; *Cg*-Gal4 > *bsk*^*DN*^, *n* = 80 nuclei). Genotypes used in (**A**-**F**) were *Cg*-Gal4 > *w*^*1118*^ (control), *Cg*-Gal4 > *foi* RNAi, *Cg*-Gal4 > *bsk*^*DN*^; *foi* RNAi*, Cg*-Gal4 > *bsk*^*DN*^. Data are represented as mean ± SEM of the biological replicates. ***p < 0.001; two-tailed Student’s t-test

We subsequently examined whether the inhibition of *foi* knockdown on the DNA endoreplication was mediated by the JNK pathway. Consistent with our hypothesis, the decreased eclosion rate, the developmental defect of fat body, the reduced cell size, the smaller nuclear size, and the reduced levels of DNA synthesis of fat body cells in *foi* RNAi larvae could be rescued by expression of *bsk*^*DN*^ (Fig. 7A-F and Additional file 1: Fig. S4D-H). These data strongly indicated that *foi* knockdown resulted in DNA endoreplication defects and subsequent growth impairment through activating the JNK signaling pathway.

### The activation of *foi* knockdown on the JNK pathway is mediated by oxidative stress caused by intracellular zinc deficiency

**Fig. 8:**
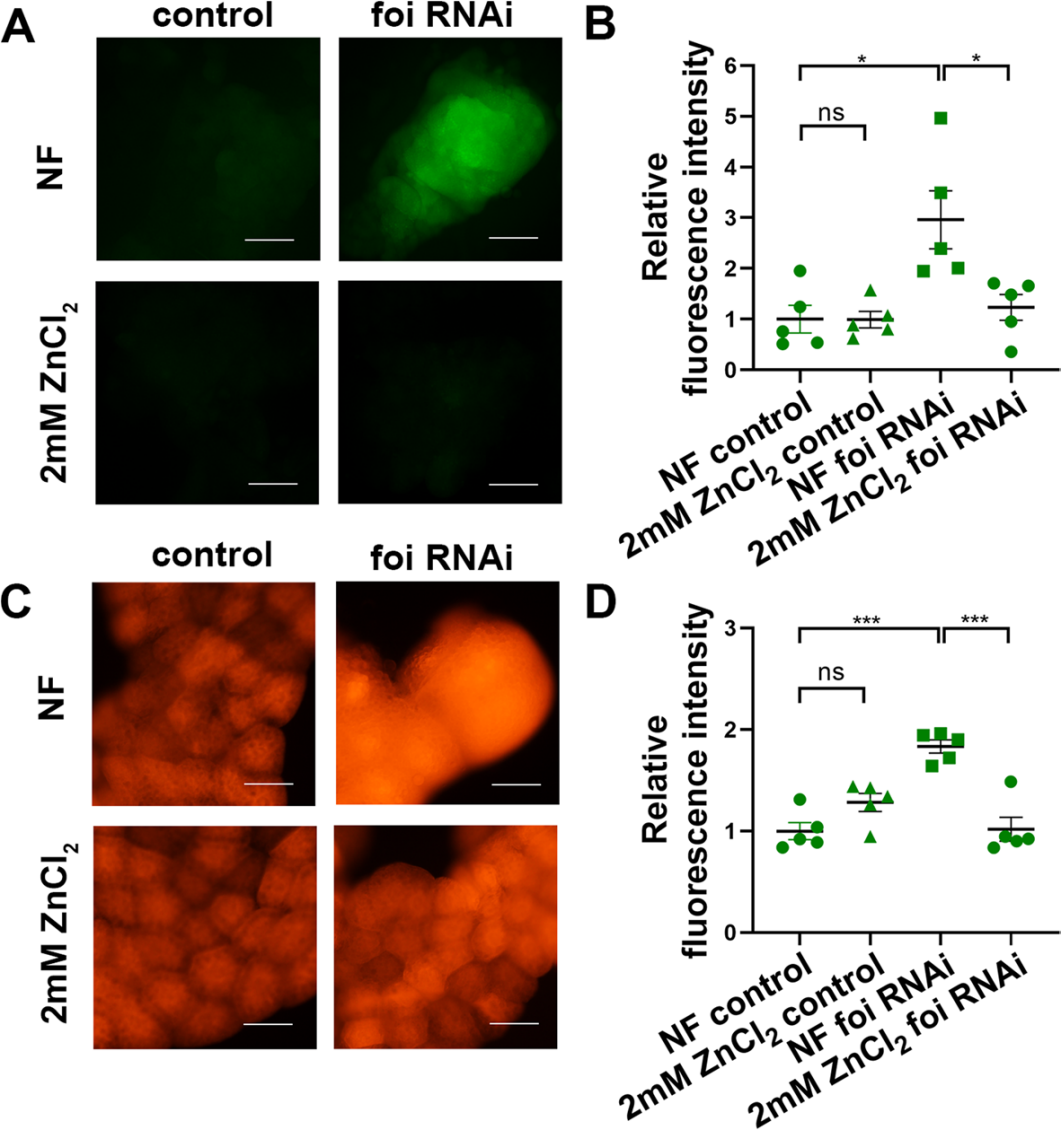
Knockdown of *foi* in the fat body leads to ROS accumulation which could be rescued by dietary zinc supplementation. **A** 20, 70-dichlorodihydro fluorescein diacetate (DCFH-DA) staining showed that *foi* RNAi in the fat body leads to ROS accumulation (green signals). Zinc supplemented in the food could rescue the ROS accumulation. *n* = 10 fat bodies per group. Scale bar, 100 μm. **B** Quantitative measurement of (**A**). *n* = 5 replicates per group. All values are presented as mean ± SEM and included in Additional file 2. **C** Dihydroethidium (DHE) method indicated that abundant ROS was generated in the fat body upon *foi* RNAi and decreased when zinc supplemented in the food. *n* = 10 fat bodies per group. Scale bar, 100 μm. **D** Quantitative measurement of (**C**). *n* = 5 replicates per group. All values are presented as mean ± SEM and included in Additional file 2. Genotypes used in (**A**-**D**) were *Cg*-Gal4 > *w*^*1118*^ (control), *Cg*-Gal4 > *foi* RNAi. Data are represented as mean ± SEM of the biological replicates. *p < 0.05; ***p < 0.001; two-tailed Student’s t-test

**Fig. 9:**
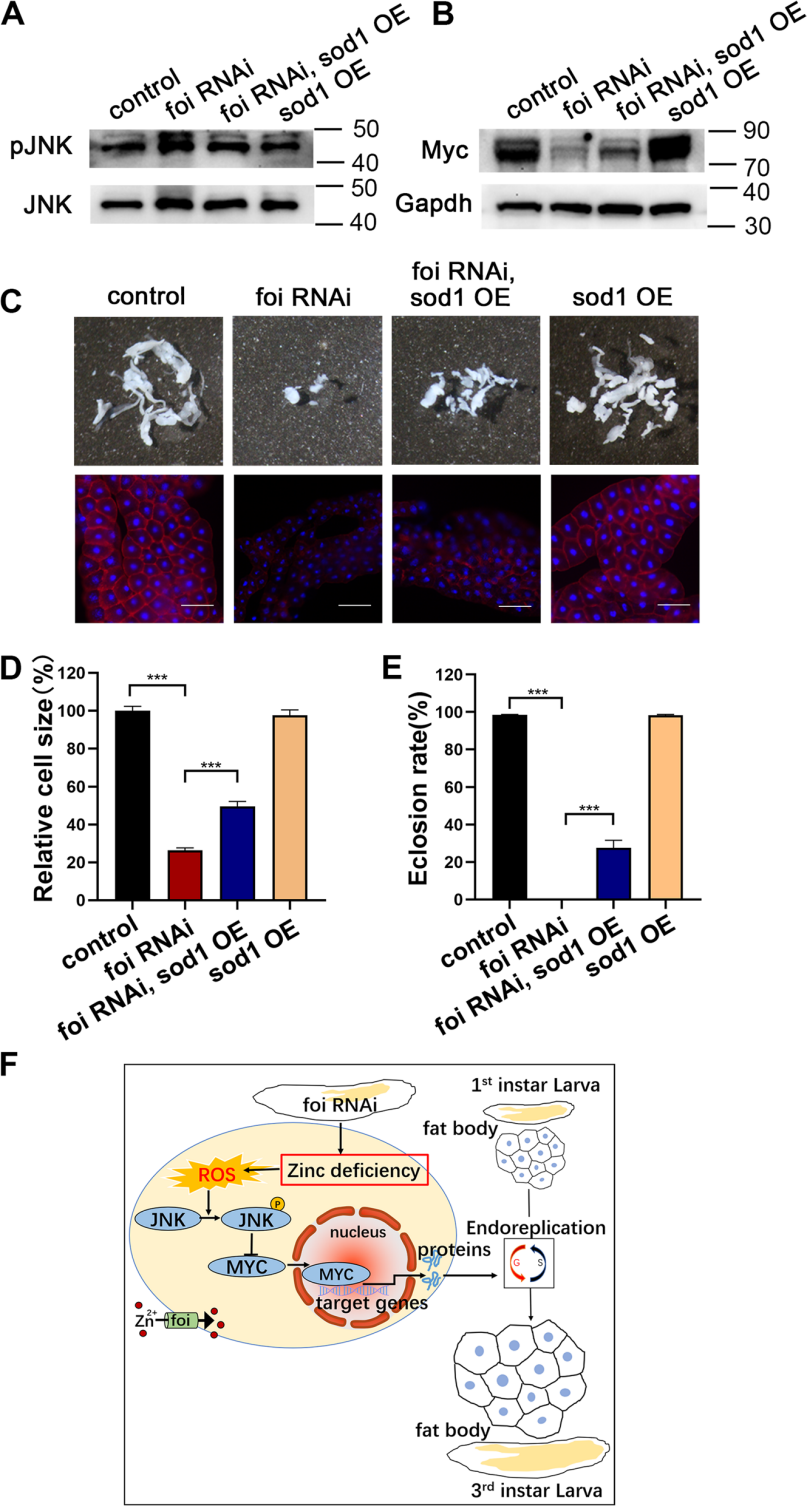
The growth arrest, fat body developmental defects and blocked endoreplication in *foi* RNAi could be rescued by *sod1* OE. **A** The increased expression of pJNK in *Cg*-Gal4 > *foi* RNAi larvae fat body was partially suppressed by *sod1* OE. *n* = 40 fat bodies per group. **B** The decreased expression of Myc in *foi* RNAi larvae fat body could be significantly restored by *sod1* OE. *n* = 40 fat bodies per group. **C** The smaller fat body size and cell size of *Cg*-Gal4 > *foi* RNAi were rescued by *sod1* OE. *n* = 6 replicates per group. Scale bar, 100 μm. **D** Quantitative measurement of the fat body cell sizes in (**C**). (control, *n* = 104; *Cg*-Gal4 > *foi* RNAi, *n* = 84; *Cg*-Gal4 > *foi* RNAi, *sod1* OE, *n* = 101; *Cg*-Gal4 > *sod1* OE, *n* = 97 cells). **E** The eclosion defect of *Cg*-Gal4 > *foi* RNAi was significantly rescued by *sod1* OE. *n* = 50–70 larvae per vial, *n* = 6 vials per experimental group. Genotypes used in (**A**-**E**) were *Cg*-Gal4 > *w*^*1118*^ (control), *Cg*-Gal4 > *foi* RNAi, *Cg*-Gal4 > *foi* RNAi, *sod1* OE, *Cg*-Gal4 > *sod1* OE. Data are represented as mean ± SEM of the biological replicates. ***p < 0.001; two-tailed Student’s t-test. **F** A model to explain the effect of zinc transporter FOI on *Drosophila* fat body endoreplication. Zinc transporter *foi* knockdown resulted in zinc deletion in the cytoplasm, leading to ROS accumulation and JNK activation, and subsequently inhibited the expression of Myc to block the DNA endoreplication in the fat body cell nucleus, then leading to fat body growth defect

The experiments above showed that the zinc deficiency in *foi* RNAi stimulates the JNK signaling pathway. We previously reported that zinc dyshomeostasis in the Golgi apparatus could stimulate the JNK signaling pathway [46]. However, the mechanisms of how zinc deficiency in the cytoplasm of *foi* RNAi modulates JNK signaling are still unclear. JNK (also known as stress-activated protein kinase) could be activated by many environmental stimuli, such as UV radiation, heat shock, oxidative stress, and so on [47]. Among them, reactive oxygen species (ROS)-mediated JNK activation is a critical component in deciding the fate of cells in response to various environmental stresses [47]. More and more research suggests that cellular zinc dyshomeostasis, either cellular zinc accumulation or deficiency, increases ROS production and dysregulated metabolic function [48]. Therefore, we wondered whether the activation of the zinc deficiency in *foi* RNAi on JNK signaling is mediated by ROS. To explore the mechanism behind *foi* knockdown-induced JNK activation, we examined the involvement of oxidative stress in the process. As shown in Fig. 8A and B, oxidation-sensitive dye, 20, 70-dichlorodihydro fluorescein diacetate (DCFH-DA) staining suggested that *foi* knockdown indeed led to abundant ROS in the fat body. The increased amount of ROS was decreased by dietary zinc supplementation (Fig. 8A-B), suggesting that zinc deficiency is responsible for the generation of ROS in *foi* RNAi. The results were further confirmed by the Dihydroethidium (DHE) method (Fig. 8C-D). Then we wondered whether the ROS clearance could mitigate the defects caused by *foi* RNAi. Superoxide dismutase (SOD) is one of several significant proteins that regulate the removal of superoxide. The *Drosophila* gene *sod1* encodes the canonical cytoplasmic Cu/Zn SOD enzyme [49, 50]. We showed that the activation of JNK and reduced Myc levels in *foi* RNAi were suppressed by overexpression of *sod1* (Fig. 9A-B, Additional file 3). Consistently, the reduced fat body and cell size of *foi* RNAi were notably restored by *sod1* OE (Fig. 9C-D and Additional file 1: Fig. S5A-B). In line with the above results, the decreased eclosion rate of *foi* RNAi was also markedly alleviated by *sod1* OE (Fig. 9E and Additional file 1: Fig. S5C). Overall, these data demonstrated that the physical defects observed in *foi* knockdown arose from the oxidative stress induced by zinc deficiency.

## Discussion

Insect fat body plays essential roles in sensing nutritional conditions, secreting proteins and peptides, and responding with the synthesis and release of energy, just like mammalian adipose tissues and the liver [51–53]. So the fat body tissues function as major sites for nutrient storage, energy metabolism, innate immunity, detoxification, and even the development and behavior of the entire insect organism [5]. DNA endoreplication during the larval stages is a critical event in fat development [31]. Here we showed that *Drosophila* zinc transporter fear-of-intimacy (*foi,* a homolog to human Zip6 and Zip10) is required for larval growth and fat body endoreplication. Fat body-specific knockdown of *foi* results in severe growth defect and arrest, which is accompanied by defects of DNA endoreplication in fat body cells. Further studies indicated that the DNA endoreplication defects arise from the decreased expression of Myc, which is mediated by the ROS-JNK signaling pathway. All phenotypes, including the growth arrest, the smaller fat body cell size, the decreased expression of Myc and the hyperactive ROS-JNK signaling pathway caused by *foi* knockdown, could be rescued by increasing intracellular zinc levels. These data suggest that FOI-mediated zinc transport is required for *Drosophila* fat body DNA endoreplication associated with the ROS-JNK signaling pathway (Fig. 9F).

We previously reported that FOI controls fat body cell dissociation during metamorphosis by modulating *Drosophila*'s Mmp activity [29]. When *foi* was modulated by *Lsp2*-Gal4, which initiates *foi* expression in the fat body cells at the mid-instar transition midway through the third larval instar, the progression of fat body cell dissociation was delayed by fat body-specific *foi* knockdown. At the same time, it was accelerated by *foi* overexpression (OE) [29]. But the underlying mechanisms of FOI function in earlier larvae fat body development remains unknown. We showed that FOI is required for fat body DNA endoreplication during the larval stages. These data suggest that FOI is critical in fat body growth during larval stages and remodeling during metamorphosis.

Our study is consistent with a previous study that *Drosophila* TRPM gene (dTRPM, a subfamily of transient receptor potential proteins) deficiency leads to a profound reduction in larval growth resulting from a decrease in cell size, and these phenotypes arose from reduced zinc levels and could be rescued by extracellular zinc supplementation [54]. Here we further identified the underlying mechanisms of zinc homeostasis regulated by FOI function in fat body endoreplication during *Drosophila* larval development. A growing body of evidence indicates that zinc deficiency induces oxidative stress and damage [55, 56]. It is known that the JNK signaling cascade is a mitogen-activated protein kinase (MAPK) signaling pathway that can be activated in response to a wide range of environmental stimuli [57]. Many diseases, such as neurodegenerative disorders, cancer, diabetes, cardiovascular diseases, and immune system disorders, are associated with the JNK signaling pathway activated by oxidative stress [58–61]. Here, we provide evidence for the role of the ROS-JNK pathway in fat body growth defects caused by zinc deficiency when *foi* was knocked down. However, this does not preclude other pathways from participating in fat body developmental defects in *foi* RNAi.

FOI involves the morphogenesis of many tissues, such as gonad, trachea and muscles in *Drosophila* [25, 27, 28]. Besides, the function of Zip6 and Zip10 in various systems has also been reported. For example, Zip6 and Zip10 are highly abundant, maternally-derived zinc transporters in the oocyte [21]. Zip10 could form a heteromer with Zip6 which regulates cell-autonomous migration responsible for gastrulation and anterior–posterior axis in zebrafish embryos [22]. In addition, Zip10 is essential for immune system development and epidermis formation in mice [17, 62]. These findings reveal crucial roles of FOI and its homologs in the development processes of vertebrates and invertebrates. Whether these processes share the same mechanism linking to the JNK signaling pathway remains further studied. Moreover, FOI controls *Drosophila* myogenesis by regulating the activity of specific zinc finger transcription factors (ZFTFs) [28]. The possible involvement of ZFTFs in the effect of FOI on the JNK signaling pathway, Myc protein and fat body development needs to be further studied.

Loss-of-function *foi* alleles are embryonic lethal, so we study the function of *foi* by RNA interference technology (RNAi). RNAi is the most efficient tool for targeted gene silencing [63]. *Drosophila* RNAi libraries have been routinely utilized to study gene functions in multiple biological disciplines. However, RNAi-based technology has many limitations in performing functional genetic screens in vivo, such as the off-target effect and incompleteness of knockdowns, especially the Vienna *Drosophila* RNAi Center (VDRC) KK RNAi lines [64]. We performed the study with two RNAi lines to avoid observing off-target effects. The *foi* RNAi (V10102, used in the main text) is a GD stock, and the *foi* RNAi 2# (V330251, used in the Supplement Material) is a shRNA stock. They target different parts of the *foi* gene. Moreover, these two different lines exhibited broadly similar phenotypes. Based on these two points, we concluded that the phenotypes we observed are caused by *foi* knockdown.

Organ size is controlled by coordinately regulating cell growth and proliferation. Dynamic zinc fluxes are essential for meiotic progression in the oocytes of several animals [65]. Besides, some zinc-finger proteins have been reported to be required for mitosis or meiosis in oocytes or spermatocytes [65–67]. We found that the initial fat body cell mitosis is also affected by *foi* knockdown (data not shown), but the underlying mechanisms need investigation. To disentangle mitosis from endoreplication, we did a temperature shift once mitosis is finished/before growth endoreplication starts [32] in *Cg*-Gal4, *tub*-Gal80^ts^ > *foi* RNAi and *Cg*-Gal4, *tub*-Gal80^ts^ > *foi* RNAi 2# (Additional file 1: Fig. S6). The results showed that the fat body cell size, the fat body size and eclosion rate were decreased in *Cg*-Gal4, *tub*-Gal80^ts^ > *foi* RNAi and *Cg*-Gal4, *tub*-Gal80^ts^ > *foi* RNAi 2# (Additional file 1: Fig. S6A-C). These results confirmed that the fat body cell endoreplication is indeed affected by *foi* knockdown.

Endoreplication provides a mechanism to enhance gene expression by increasing the availability of DNA templates. Thus, it generally occurs in tissues with high metabolic activity, such as salivary glands, fat body, dipterans' silk glands, and placental trophoblast giant cells of rodents [42, 68]. Since zinc homeostasis is critical for fat body DNA endoreplication in *Drosophila*, dietary zinc administration may provide a new therapeutic target for human diseases related to endoreplication.

## Conclusions

In summary, we have demonstrated the function of FOI in the DNA endoreplication of the *Drosophila* fat body. By genetically manipulating zinc transporters' expression in the fat body or regulating the dietary zinc intake, we can modify the phenotypes of *foi* knockdown larvae. FOI affects DNA endoreplication by modulating Myc, and the ROS-JNK signaling pathway mediates this process. The developmental defects and decreased Myc levels caused by *foi* knockdown could be restored by reducing ROS or inhibiting the activity of the JNK signaling pathway. Finally, our report highlights the need to investigate zinc homeostasis further and should motivate future studies to understand the interactions of zinc with other pathways during development. Understanding the molecular mechanisms responsible for DNA endoreplication may provide insight into potential therapeutic strategies for many human diseases.

## Methods

### *Drosophila* strains and culture media

Unless otherwise noted, flies were maintained at 25 °C with 60% humidity in traditional corn-yeast food. For the experiments where *Cg*-Gal4, *tub-*Gal80^*ts*^ was used, parental flies were left to lay eggs on a fresh culture vial for 1 day at a restrictive temperature (18 °C). We considered the time of removing the flies from the vial 12 ± 12 h after egg laying [32]. Cultures were maintained for three days at 18℃ after egg laying, followed by transfer of cultures (first instar larval stages) to 29℃ and dissection three days later (third instar larval stages) [69].

The concentrations of supplemented metals, metal chelators and reagents used were as follows: 2 mM ZnCl_2_ (Sigma, Cat#746,355), 25 μM zinc chelator N,N,N′,N′-tetrakis (2-pyridylmethyl) ethylenediamine (TPEN) (Sigma, Cat#P4413), 5 mM ferric ammonium citrate (FAC) (Sigma, Cat#F5879), 200 μM iron-specific chelator bathophenanthrolinedisulfonic acid disodium (BPS) (Sigma, Cat#146,617), 2 mg/mL Chloroquine (CQ) (Sigma, Cat#6628). *Cg-Gal4* (Bloomington #7011), *UAS-p35* (Bloomington #5072), *bsk*^*DN*^ (Bloomington #6409), *UAS-sod1* (Bloomington #24,750) were obtained from the Bloomington *Drosophila* Stock Center. *w*^*1118*^ (VDRC#60,000), *foi RNAi* (VDRC#10,102), and *foi RNAi* 2# (VDRC#330,251) were obtained from the Vienna *Drosophila* RNAi Center. One line with more severe phenotypes (VDRC#10,102) was chosen for the studies reported here. *UAS-foi* was kind gifts from Dr. Mark Van Doren [25]. *UAS-Myc*^*42*^ and *UAS-Myc*^*132*^ were kindly provided by Dr. Lei Xue [70]. *UAS-dZnT1* [71] and *UAS-dZip1* [72] were kind gifts from Dr. Bing Zhou.

### Eclosion assays

*Cg*-Gal4 was crossed to wild-type, *foi* RNAi, *foi* OE or other transgenic flies on juice-agar plates. Newly hatched progeny were transferred to normal food (NF) or food supplemented with different metals or metal chelators, as indicated in each experiment. The density of each vial was controlled to about 50–70 larvae, and the total number of emerged adults of each genotype was counted. Six parallel group tests were conducted for each genotype, and the experiments were repeated thrice**.**

### Alkaline phosphatase (ALP) activity assay

Samples (fat bodies of 40 third instar larvae) were lysed in ALP lysis buffer (1.0 mM Tris–HCl pH7.4, 0.5 mM MgCl_2_, and 0.1% Triton X-100), then 90 μl solution A (1.0 M diethanolamine, 0.5 mM MgCl_2_ pH9.8) and 10 μl solution B (150 mM p-nitrophenyl phosphate) were added. The absorbance at 405 nm was measured after incubation for 30 min at 25 °C [73]. Protein concentration was measured by the BCA kit (Thermo Scientific, 23,227). Equal amounts of the total proteins were assayed in each group. The experiments were repeated at least three times**.**

### 5-Ethynyl-2'-deoxyuridine (EdU) staining

Larvae were fed EdU at 100 ug/ml from 72 to 96 h after egg deposition. After that, fat bodies were dissected in PBS, fixed with 4% paraformaldehyde for 10 min and permeated with PBST for 5 min three times. Afterwards, the fat body was incubated with the Click Reaction Mixture for 30 min at room temperature in a dark place and then incubated with Hoechst 33,342 for 10 min using an EdU kit (BeyoClick™ EdU Cell Proliferation Kit with Alexa Fluor 488, Beyotime, China). The samples were observed by a Nikon Ti2 fluorescence microscope. The EdU intensity and nuclear area were measured with ImageJ software (US National Institutes of Health). The relative EdU signals were shown as EdU intensity/nuclear area, and about 42–108 nuclei were analyzed per genotype. *n* = 6 replicates per group. The experiment was repeated at least three times.

### Morphological and cellular analyses

For the larval and pupal morphology analysis, the larvae or pupae were collected at 25℃ and frozen to death at -80℃ for one night. The larvae or pupae were photographed using a Nikon camera stereomicroscope. The pupal sizes were measured with ImageJ software after photographing. More than ten larvae or pupae were scored per genotype. The experiment was repeated at least three times.

For the fat body, cell and nuclear size, the third instar larvae fat bodies were dissected in cold PBS within 20 min and fixed in 4% paraformaldehyde for 20 min. The fat bodies were photographed using a Nikon camera for the fat body morphology analysis. For the cell and nuclear size assay, the third instar larvae fat bodies were dissected, fixed and washed in PBST, after which they were incubated in 100 nM Phalloidin (yeasen, 40734ES75) in PBS for 30 min and then washed three times in PBST and stained with 4’,6-diamidino-2-phenylindole (DAPI, Beyotine, C1005). After several washes, fat bodies were sorted and mounted in 50% glycerol/PBS. The fat bodies were photographed using a Nikon Ti2 fluorescence microscope. Cell and nuclear size were then measured with ImageJ software. About 88–162 cells were scored per genotype. The experiment was repeated at least three times.

### RNA isolation, semiquantitative RT-PCR, and quantitative real-time PCR

The total RNA of 40 third-instar larvae fat bodies was extracted using the TRIzol reagent (Invitrogen). cDNA was reverse-transcribed from 1–1.5 μg total RNA with EasyScript One-Step gDNA Removal and cDNA Synthesis SuperMix (TransGen) according to the manufacturer’s instructions. Real-time PCR was performed using PerfectStart Green qPCR SuperMix (TransGen). Samples were normalized using *rp49* primers. The primers used for PCR were:

- MtnB Fw, ATCAGTTCGCCTCAGCCAAG;
- MtnB Rv, GCAAACGCACTGGCAATCCT;
- rp49 Fw, GCACCAAGCACTTCATCC;
- rp49 Rv, CGATCTCGCCGCAGTAAA.

### Western blot analysis

Fat bodies of 40 third instar larvae were extracted with PBST containing protease inhibitors (APExBIO, K1007) and phosphatase inhibitor cocktail (Beyotine, P1081), and the protein concentration was measured by the BCA kit (Thermo Scientific, 23227). Equal amounts of total protein were subjected to western blotting [42]. Fat body samples were separated by SDS-PAGE in 10% gels and transferred to PVDF membranes (Millipore). The primary antibodies used were rabbit anti-pJNK (Millipore, 07–175, 1:2000), anti-JNK (zen-bio, AF6318, 1:1000), anti–Myc (DSHB, P4C4-B10, 1:100) and anti-GADPH (Servicebio, GB11002, 1:2000). Secondary peroxidase–conjugated antibodies used were goat anti-mouse (BOSTER, BA1054, 1:5000) and goat anti-rabbit (BOSTER, BA1050, 1:5000). Signals were developed with an ECL detection kit (TransGen, DW101-01). The images were acquired with a gel documentation system (ProteinSimple, FluorChem E). The signals were measured with ImageJ software. All the experiments were repeated at least three times.

### ROS detection

Oxidation-sensitive dyes were used as fluorescence agents to detect ROS in the fat body. Fat bodies were dissected in cold PBS, fixed and washed with PBS. The tissues were then incubated with 20, 70-dichlorodihydro fluorescein diacetate (DCFH-DA, Sigma, Cat#2044–85-1) for about 10 min in a dark chamber. The tissues were washed with PBS and quickly mounted with 50% glycerol (diluted with PBS). Then the samples were imaged with a fluorescence microscope (Nikon Ti2, Japan). Dihydroethidium (DHE)(ThermoFisher, Cat#D11347) method was performed as previously reported (www.nature.com/protocolexchange/protocols/414#/reagents). For DCFH-DA and DHE quantitative assays, the relative fluorescence intensity means fluorescence intensity/tissue area. Six parallel group tests were conducted for each genotype, and all the experiments were repeated thrice.

### Statistical analysis

Data were analyzed with GraphPad Prism 8 (La Jolla, CA, USA). All data were analyzed by Student t-tests. Statistical results were presented as means ± SEM. Asterisks indicate critical levels of significance (* *P* < 0.05, ** *P* < 0.01, and *** *P* < 0.001).

## Supplementary Information

**Additional file 1.** foi RNAi 2# related and other supplemented data. **Figure Sl.** Fat body development defects caused by *foi* RNAi 2# (V330251#) are largely similar with *foi* RNAi (V10102#). Related to Figs. 1, 2 and 3. **Figure S2.** The reduced fat body size of *Cg*-Gal4 > *foi* RNAi larvae could be exacerbated by TPEN or *dZnT1* OE. Related to Figs. 2 and 3. **Figure S3.** Sensitivity Study of TPEN on the fat body development of wild-type Drosophila. Related to Fig. 2. **Figure S4.** The growth arrest, fat body developmental defects and blocked endoreplication in *foi* RNAi 2# could be rescued by JNK signaling inhibition. Related to Figs. 5, 6 and 7. **Figure S5.** The fat body developmental defects and growth arrest of *foi* RNAi 2# could be rescued by *sod1* OE. Related to Fig. 9. **Figure S6.** *Drosophila * FOI is required for larval fat body development.

**Additional file 2.** Raw data for graphs with *n* < 6.

**Additional file 3.** Uncropped blots.

## Data Availability

All data generated or analysed during this study are included in this published article and its supplementary information files.

## Abbreviations

ALP: Alkaline phosphatase
BPS: Bathophenanthrolinedisulfonic acid disodium
CQ: Chloroquine
EdU: 5-Ethynyl-2'-deoxyuridine
FAC: Ferric ammonium citrate
FOI: Fear of intimacy
OE: Overexpression
TPEN: N, N, N′, N′-tetrakis (2pyridylmethyl) ethylenediamine
