# Supplementary material for: Fear-of-intimacy-mediated zinc transport is required for Drosophila fat body endoreplication
Source: BMC Biol. 2023 Apr 17;21:88. doi: 10.1186/s12915-023-01588-0 (PMC10111752; doi:10.1186/s12915-023-01588-0)
Supplement: Supplementary file 1 — Additional file 1. foi RNAi 2# related and other supplemented data. Figure Sl. Fat body development defects caused by foi RNAi 2# (V330251#) are largely similar with foi RNAi (V10102#). Related to Figs. 1, 2 and 3. Figure S2. The reduced fat body size of Cg-Gal4 > foi RNAi larvae could be exacerbated by TPEN or dZnT1 OE. Related to Figs. 2 and 3. Figure S3. Sensitivity Study of TPEN on the fat body development of wild-type Drosophila. Related to Fig. 2. Figure S4. The growth arrest, fat body developmental defects and blocked endoreplication in foi RNAi 2# could be rescued by JNK signaling inhibition. Related to Figs. 5, 6 and 7. Figure S5. The fat body developmental defects and growth arrest of foi RNAi 2# could be rescued by sod1 OE. Related to Fig. 9. Figure S6. Drosophila FOI is required for larval fat body development. [file 12915_2023_1588_MOESM1_ESM.docx]

**Additional file 1:**


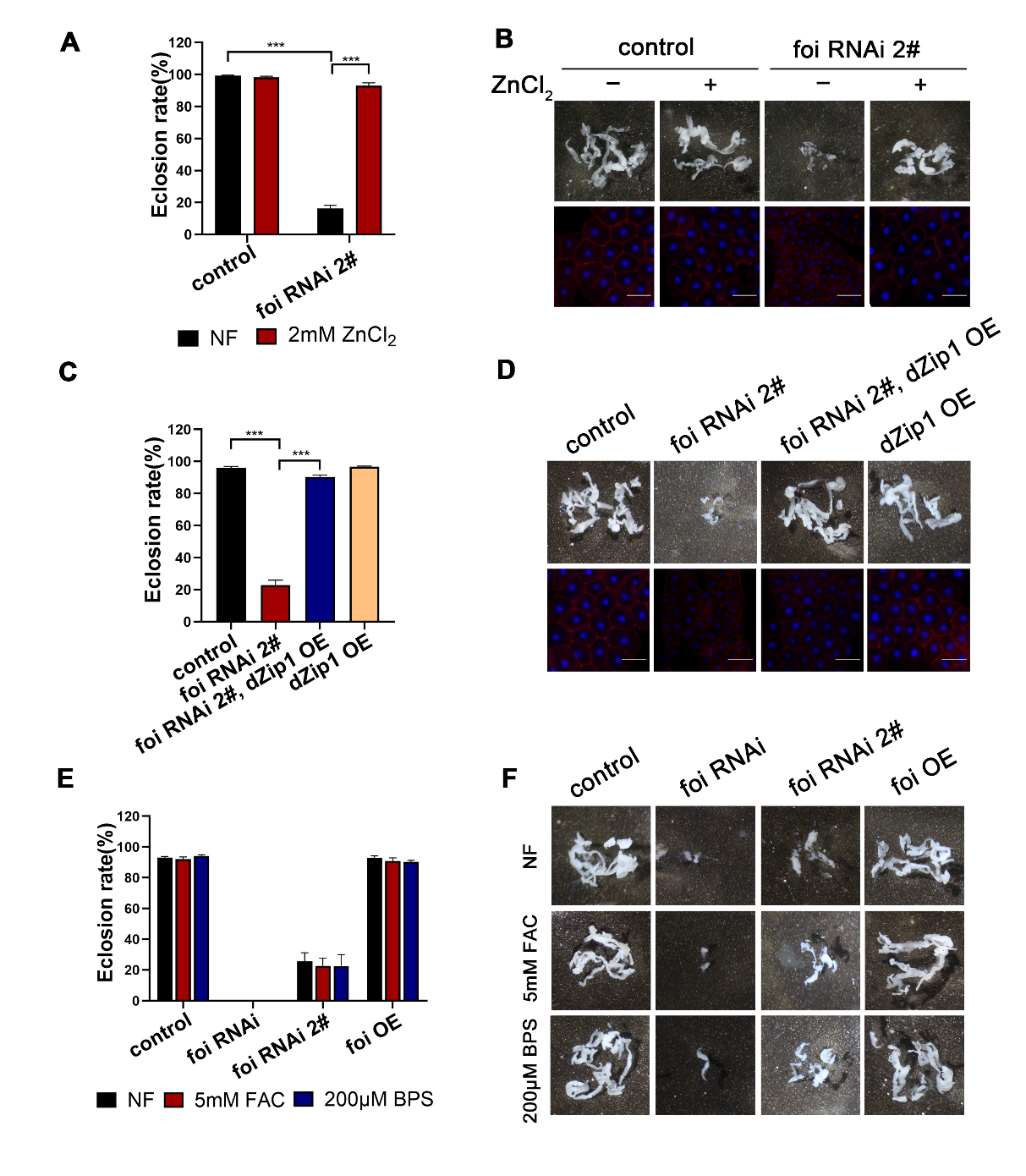


**Figure Sl. Fat body development defects caused by *foi* RNAi 2# (V330251#) are largely similar with *foi* RNAi (V10102#). Related to Figure 1-3.**

(A) The eclosion rate of *Cg*-Gal4 > *foi* RNAi 2# larvae could be rescued by dietary zinc supplementation. *Cg*-Gal4 was crossed to wild-type (*w^1118^*, control), *foi* RNAi 2# flies on juice-agar plates. Newly hatched progeny were transferred to normal food (NF), or food supplemented with 2mM ZnCl_2_. Percentages of flies that eclosed to adults were counted. n = 50-70 larvae per vial, n = 6 vials per experimental group.

(B) The smaller fat body size and cell size of *Cg*-Gal4 > *foi* RNAi 2# larvae could be rescued by dietary zinc supplementation in food. n = 6 replicates per group. Scale bar, 100 μm.

Genotypes used in (A-B) were *Cg*-Gal4 > *w^1118^* (control), *Cg*-Gal4 *> foi* RNAi 2#.

(C) The eclosion defect of *Cg*-Gal4 > *foi* RNAi 2# larvae was significantly rescued by *dZip1 OE.* n = 50-70 larvae per vial, n = 6 vials per experimental group.

(D) The reduced fat body size and cell size of *Cg*-Gal4 > *foi* RNAi 2# larvae could be significantly rescued by *dZip1* OE. n = 6 replicates per group. Scale bar, 100 μm.

Genotypes used in (C-D) were *Cg*-Gal4 > *w^1118^* (control), *Cg*-Gal4 *> foi* RNAi 2#, *Cg*-Gal4 *> foi* RNAi 2#; *dZip1* OE, *Cg*-Gal4 *> dZip1* OE.

(E) Dietary iron supplementation or iron depletion showed no significant effect on the decreased eclosion rates of *Cg*-Gal4 > *foi* RNAi and *Cg*-Gal4 > *foi* RNAi 2#. *Cg*-Gal4 was crossed to wild-type, *foi* RNAi, *foi* RNAi 2# or *foi* OE flies on juice-agar plates. Newly hatched progeny were transferred to normal food (NF), or food supplemented with 5mM FAC or 200μM BPS. Percentages of flies that eclosed to adults were counted. n = 50-70 larvae per vial, n = 6 vials per experimental group.

(F) Dietary iron supplementation and iron depletion showed no significant effect on fat body size of *Cg*-Gal4 > *foi* RNAi and *Cg*-Gal4 > *foi* RNAi 2#. n = 6 replicates per group.

Genotypes used in (E-F) were *Cg*-Gal4 > *w^1118^* (control), *Cg*-Gal4 *> foi* RNAi, *Cg*-Gal4 *> foi* RNAi 2#, *Cg*-Gal4 *> foi* OE.

Data are represented as mean ± SEM of the biological replicates. ***p < 0.001; two-tailed Student’s t-test. OE, overexpression.


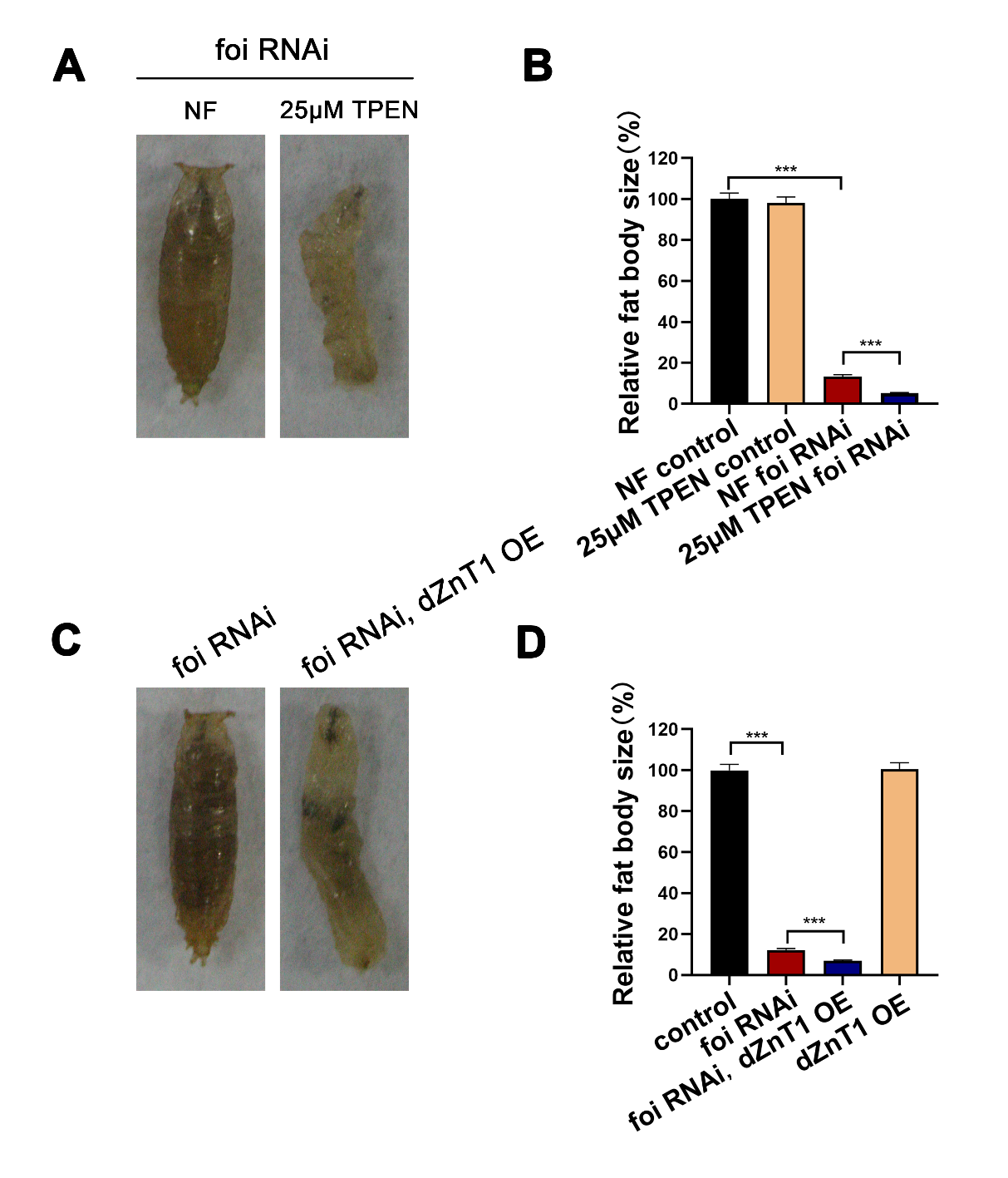


**Figure S2. The reduced fat body size of *Cg*-Gal4 > *foi* RNAi larvae could be exacerbated by TPEN or *dZnT1* OE. Related to Figure 2-3.**

(A) *foi* RNAi was lethal at the pupal stage, but *foi* RNAi cultured on TPEN were lethal at the larvae stage.

(B) The reduced fat body size of *Cg*-Gal4 > *foi* RNAi larvae could be exacerbated by 25μM TPEN. n = 10 replicates per group.

Genotypes used in (A-B) were *Cg*-Gal4 > *w^1118^* (control), *Cg*-Gal4 *> foi* RNAi

(C) *foi* RNAi was lethal at the pupal stage, while *foi* RNAi, *dZnT1* OE was lethal at the larvae stage.

(D) The reduced fat body size of *Cg*-Gal4 > *foi* RNAi larvae could be exacerbated by *dZnT1* OE. n = 10 replicates per group.

Genotypes used in (C-D) were *Cg*-Gal4 > *w^1118^* (control), *Cg*-Gal4 *> foi* RNAi, *Cg*-Gal4 *> foi* RNAi; *dZnT1* OE, *Cg*-Gal4 *> dZnT1* OE.

Data are represented as mean ± SEM of the biological replicates. ***p < 0.001; two-tailed Student’s t-test.


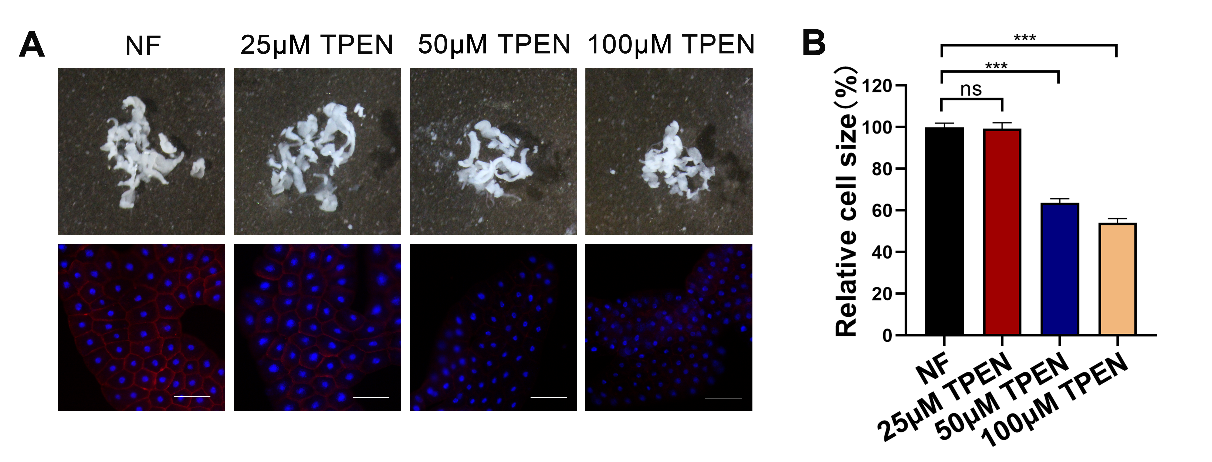


**Figure S3. Sensitivity Study of TPEN on the fat body development of wild-type Drosophila. Related to Figure 2.**

(A) 25μM TPEN did not affect the fat body development of wild type Drosophila, while more than 50μM TPEN had a serious effect on the fat body development.

(B) Quantitative measurement of the cell size in (A). (NF *w^1118^*, n = 106; 25μM TPEN *w^1118,^* n = 104; 50μM TPEN *w^1118^*, n = 91; 100μM TPEN *w^1118^*, n = 89)

Genotype used in (A-B) was *w^1118^*.

Data are represented as mean ± SEM of the biological replicates. ***p < 0.001; two-tailed Student’s t-test.


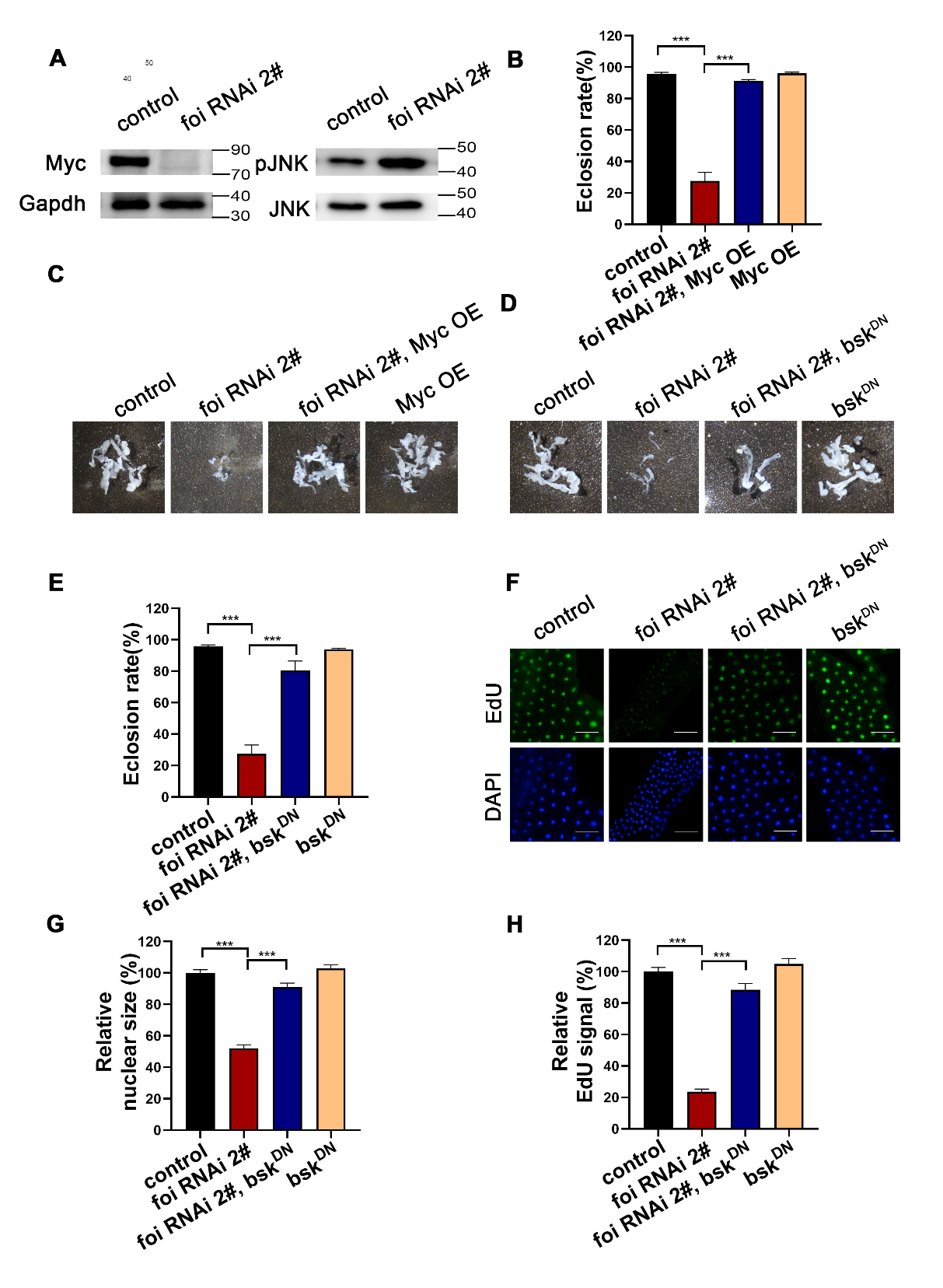


**Figure S4. The** **growth arrest, fat body developmental defects and blocked endoreplication in *foi*** **RNAi 2# could be rescued by JNK signaling inhibition. Related to Figure 5-7.**

(A) *Cg*-Gal4 > *foi* RNAi 2# showed a decreased Myc level and an increased phosphorylated JNK level in the fat body of third instar larvae in comparison to that of control. n = 40 fat bodies per group. Genotypes were *Cg*-Gal4 > *w^1118^* (control), *Cg*-Gal4 *> foi* RNAi 2#.

(B) The decreased eclosion rate of *Cg*-Gal4 > *foi* RNAi 2# was almost fully rescued by *Myc* OE. n = 50-70 larvae per vial, n = 6 vials per experimental group.

(C) The smaller fat body size of *Cg*-Gal4 > *foi* RNAi 2# was significantly rescued by *Myc* OE.

Genotypes used in (A-B) were *Cg*-Gal4 > *w^1118^* (control), *Cg*-Gal4 *> foi* RNAi 2#, *Cg*-Gal4 *> foi* RNAi 2#; *Myc OE, Cg*-Gal4 *> Myc OE*.

(D) The smaller fat body size of *Cg*-Gal4 > *foi* RNAi 2# was significantly rescued by *bsk^DN^*.

(E) The decreased eclosion rate of *Cg*-Gal4 > *foi* RNAi 2# was almost fully rescued by *bsk^DN^*. n = 50-70 larvae per vial, n = 6 vials per experimental group.

(F) The endoreplication defects observed in *Cg*-Gal4 > *foi* RNAi 2# fat body nuclei were significantly rescued by *bsk^DN^*. n = 6 replicates per group. Scale bar, 100 μm.

(G) Quantitative measurement of the nuclei size in (F). (control, n = 147; *Cg*-Gal4 > *foi* RNAi 2#, n = 61; *Cg*-Gal4 > *bsk^DN^*; *foi* RNAi 2#, n = 162; *Cg*-Gal4 > *bsk^DN^*, n = 152).

(H) Quantitative measurement of the replication signals in (F). (control, n = 108; *Cg*-Gal4 > *foi* RNAi 2#, n = 77; *Cg*-Gal4 > *bsk^DN^*; *foi* RNAi 2#, n = 109; *Cg*-Gal4 > *bsk^DN^*, n = 52).

Genotypes used in (D-H) were *Cg*-Gal4 > *w^1118^* (control), *Cg*-Gal4 *> foi* RNAi 2#, *Cg*-Gal4 *> bsk^DN^*; *foi* RNAi 2#*, Cg*-Gal4 *> bsk^DN^*.

Data are represented as mean ± SEM of the biological replicates. ***p < 0.001; two-tailed Student’s t-test. OE, overexpression.


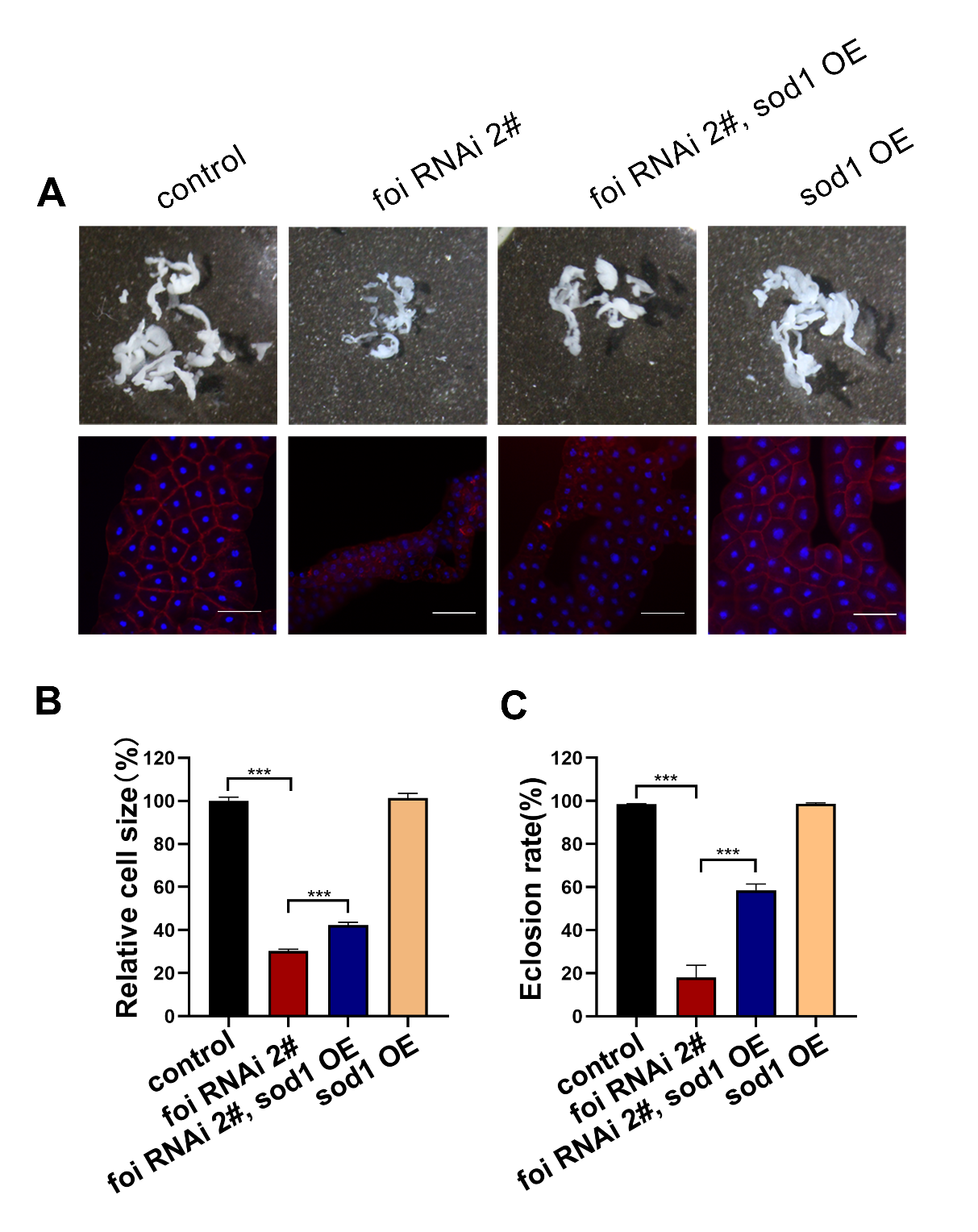


**Figure S5. The fat body developmental defects and growth arrest of *foi* RNAi 2# could be rescued by** *sod1* **OE. Related to Figure 9.**

(A) The smaller fat body size and cell size of Cg-Gal4 > *foi* RNAi 2# was rescued by *sod1* OE. n = 6–10 replicates per group. Scale bar, 100 μm.

(B) Quantitative measurement of the cell size in (A). (control, n = 100; *Cg*-Gal4 > *foi* RNAi 2#, n = 99; *Cg*-Gal4 > *foi* RNAi 2#, *sod1* OE n = 102; *Cg*-Gal4 > *sod1* OE, n = 104).

(C) The decreased eclosion rate of *Cg*-Gal4 > *foi* RNAi 2# was partially rescued by *sod1* OE. n = 50-70 larvae per vial, n = 6 vials per experimental group.

Genotypes used in (A-C) were *Cg*-Gal4 > *w^1118^* (control), *Cg*-Gal4 > *foi* RNAi 2#, *Cg*-Gal4 > *foi* RNAi 2#, *sod1* OE, *Cg-*Gal4 > *sod1* OE.

Data are represented as mean ± SEM of the biological replicates. ***p < 0.001; two-tailed Student’s t-test.


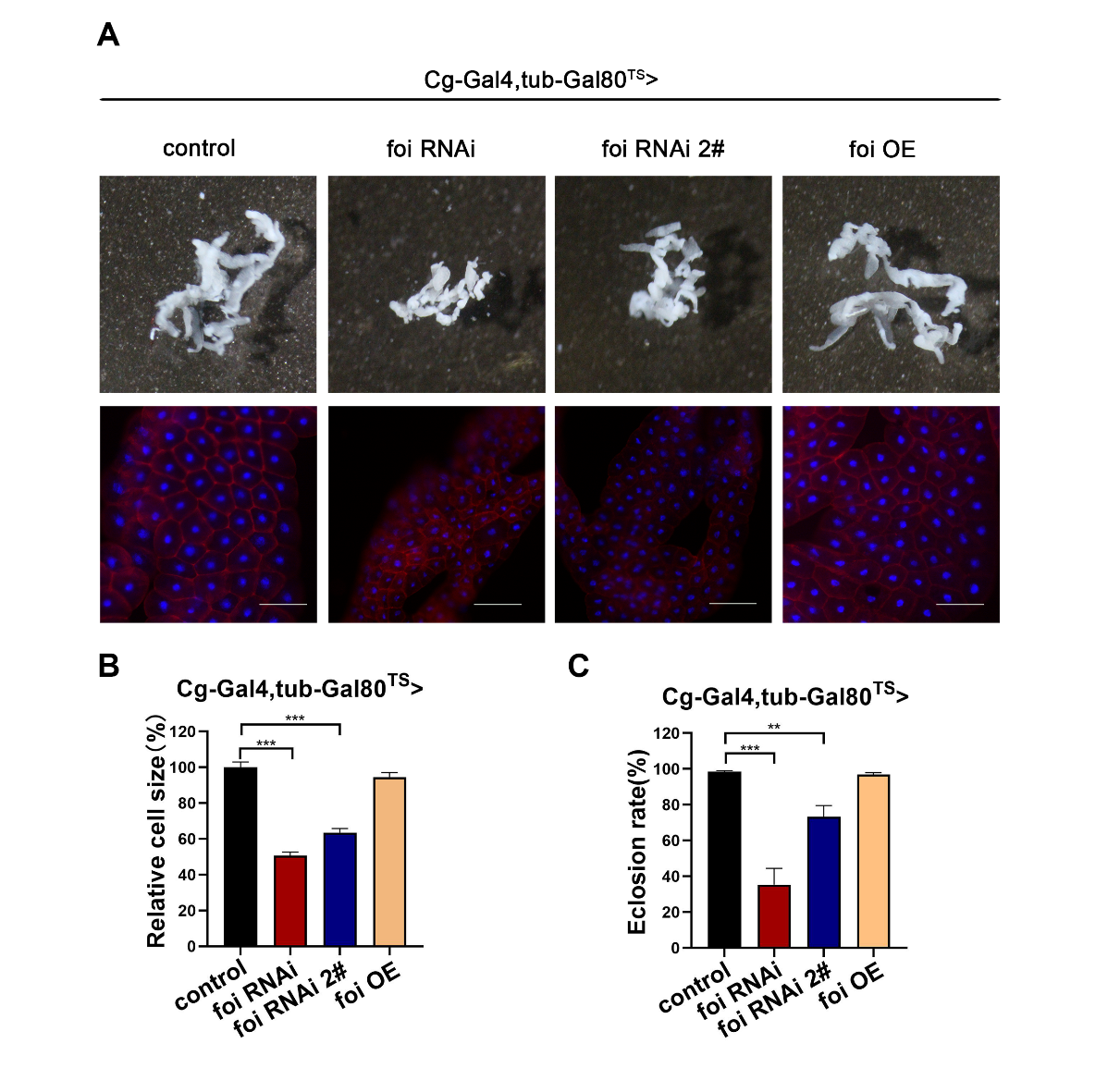


**Figure S6. *Drosophila foi* is required for larval fat body development.**

(A) Knockdown of *foi* once mitosis is finished / before growth endoreplication starts led to reduced fat body size and cell size in the fat body. Genotypes used are *Cg*-Gal4, *tub*-Gal80^TS^ > *w^1118^* (control), *Cg*-Gal4, *tub*-Gal80^TS^ *> foi* RNAi, *Cg*-Gal4, *tub*-Gal80^TS^ *> foi* RNAi 2# or *Cg*-Gal4, *tub*-Gal80^TS^ *> foi* OE. n = 6 replicates per group. Scale bar, 100 μm.

(B) Quantitative measurement of the fat body cell sizes in (A). (control, n = 99; *Cg*-Gal4, *tub*-Gal80^TS^ *> foi* RNAi n = 102; *Cg*-Gal4, *tub*-Gal80^TS^ *> foi* RNAi 2# n = 102; *Cg*-Gal4, *tub*-Gal80^TS^ *> foi* OE, n = 103).

(C) Knockdown of *foi* once mitosis is finished / before growth endoreplication starts resulted in a reduced eclosion rate. n = 50-70 larvae per vial, n = 6 vials per experimental group. Genotypes used are *Cg*-Gal4, *tub*-Gal80^TS^ > *w^1118^* (control), *Cg*-Gal4, *tub*-Gal80^TS^ *> foi* RNAi, *Cg*-Gal4, *tub*-Gal80^TS^ *> foi* RNAi 2# or *Cg*-Gal4, *tub*-Gal80^TS^ *> foi* OE. n≥3. **p < 0.01, ***p < 0.001; two-tailed Student’s t-test. OE, overexpression.
